# Plasma membrane damage repair is mediated by an acid sphingomyelinase in *Entamoeba histolytica*

**DOI:** 10.1371/journal.ppat.1008016

**Published:** 2019-08-28

**Authors:** Fátima Ramírez-Montiel, Claudia Mendoza-Macías, Sairy Andrade-Guillén, Ángeles Rangel-Serrano, Itzel Páramo-Pérez, Paris E. Rivera-Cuéllar, B. Liliana España-Sánchez, Gabriel Luna-Bárcenas, Fernando Anaya-Velázquez, Bernardo Franco, Felipe Padilla-Vaca

**Affiliations:** 1 Departmento de Biología, División de Ciencias Naturales y Exactas, Universidad de Guanajuato, Guanajuato, Guanajuato, Mexico; 2 Departmento de Farmacia, División de Ciencias Naturales y Exactas, Universidad de Guanajuato, Guanajuato, Guanajuato, Mexico; 3 CONACYT_Centro de Investigación y Desarrollo en Electroquímica (CIDETEQ) S.C. Parque Tecnológico, San Fandila, Querétaro, México; 4 Centro de Investigación y de Estudios Avanzados del Instituto Politécnico Nacional (CINVESTAV) Unidad Querétaro, Fracc. Real de Juriquilla, Querétaro, Querétaro, México; University of Virginia, UNITED STATES

## Abstract

*Entamoeba histolytica* is a pathogen that during its infective process confronts the host defenses, which damages the amoebic plasma membrane (PM), resulting in the loss of viability. However, it is unknown whether amoebic trophozoites are able to repair their PM when it is damaged. Acid sphingomyelinases (aSMases) have been reported in mammalian cells to promote endocytosis and removal of PM lesions. In this work, six predicted amoebic genes encoding for aSMases were found to be transcribed in the HM1:IMSS strain, finding that the *EhaSM6* gene is the most transcribed in basal growth conditions and rendered a functional protein. The secreted aSMase activity detected was stimulated by Mg^+2^ and inhibited by Co^+2^. Trophozoites that overexpress the *EhaSM6* gene (HM1-SM6HA) exhibit an increase of 2-fold in the secreted aSMase activity. This transfectant trophozoites exposed to pore-forming molecules (SLO, Magainin, β-Defensin 2 and human complement) exhibited an increase from 6 to 25-fold in the secreted aSMase activity which correlated with higher amoebic viability in a Ca^+2^ dependent process. However, other agents that affect the PM such as hydrogen peroxide also induced an increase of secreted aSMase, but to a lesser extent. The aSMase6 enzyme is N- and C-terminal processed. Confocal and transmission electron microscopy showed that trophozoites treated with SLO presented a migration of lysosomes containing the aSMase towards the PM, inducing the formation of membrane patches and endosomes in the control strain. These cellular structures were increased in the overexpressing strain, indicating the involvement of the aSMase6 in the PM injury repair. The pore-forming molecules induced an increase in the expression of *EhaSM1*, *2*, *5* and *6* genes, meanwhile, hydrogen peroxide induced an increase in all of them. In all the conditions evaluated, the *EhaSM6* gene exhibited the highest levels of induction. Overall, these novel findings show that the aSMase6 enzyme from *E*. *histolytica* promotes the repair of the PM damaged with pore-forming molecules to prevent losing cell integrity. This novel system could act when encountered with the lytic defense systems of the host.

## Author summary

The host-amoeba relationship is based on a series of interplays between host defense mechanisms and parasite survival strategies. While host cells elaborate diverse mechanisms for pathogen elimination, *Entamoeba histolytica* trophozoites have also developed complex strategies to counteract host immune response and facilitate its own survival while confronting host defenses. *E*. *histolytica* exposed to pore-forming proteins such as β-Defensin 2, human complement and Streptolysin O (SLO), increases the activity of secreted aSMase, which is related to greater amoebic viability. Other agents that affect plasma membrane (PM) may also increase secreted aSMase but to a lesser extent. SLO form pores in the PM of *E*. *histolytica* trophozoites that initiates the uncontrolled entry of Ca^2+^, recognized as the primary trigger for cell responses which favors the migration of the lysosomes to the periphery of the cell, fuses with the PM and release their content, including aSMase to the external side of the cell. The secreted aSMase favoring the internalization of the lesion for its degradation in phagolysosomes. During the early stages of PM damage, the pores are rapidly blocked by patch-like structures that prevent the lysis of the trophozoite and immediately begin internalizing the lesion. The aSMase6 overexpression favors the repair of the lesion and the survival of *E*. *histolytica* trophozoites. Pore-forming proteins induced an increase in the expression of *EhaSM1*, *2*, *5* and *6* genes, meanwhile oxidative stress induced an increase in all of them. Here we report, for the first time, that *E*. *histolytica* possess a mechanism for PM damage repair mediated by aSMase similar to the system described in mammalian cells.

## Introduction

It is well established that the plasma membrane (PM) is the most important component of the cell that maintains its integrity and homeostasis during physiological changes. However, its integrity is regularly infringed by many mechanical or biochemical factors that endanger cell viability. The eukaryotic cells have developed mechanisms that help repair injuries and prevent the release of cytoplasmic components, thus restoring complete functionality or allowing their elimination through apoptosis [1,2]. The first sign of damage to the PM is the uncontrolled entry of Ca^+2^, which activates different mechanisms in order to repair the damaged membrane [1,3].

The repair of injuries by a spontaneous reorganization of membrane phospholipids can only occur if the membrane damage is not recurrent and the lesion is under 0.2 μm in diameter [3,4]. More frequent or larger lesions need the replacement of the damaged area by the elimination of the lesion for which two main mechanisms have been described. One repair mechanism is mediated by the annexins which are Ca^2+^ sensors that merge at the site of damage in the PM and promote its elimination [4–7]. Another repair mechanism involves the exocytosis of lysosomal acid sphingomyelinase (aSMase) that can trigger the formation of endosomes that internalize the lesion regenerating the integrity of the PM [7–9]. After damage to the PM and the uncontrolled entry of Ca^2+^, there is a recruitment of lysosomes to the site of the lesion and then they fuse to the PM, releasing the aSMase which hydrolyzes sphingomyelin into ceramide; this, in turn, favors the formation of endosomes that internalize the lesion and restores the integrity of the membrane [8–11].

Intestinal amoebiasis is a parasitic infection that affects humans and is caused by the protozoan *Entamoeba histolytica*, resulting in 40,000 to 100,000 deaths annually worldwide [12–14]. *E*. *histolytica* infection is a multifactorial process in which adaptation is the key for parasite survival, which is dictated by specific molecules and the mechanisms to confront the host immune system. The first line of innate immune defense which amoebae confront is the mucus layer that acts as a protective barrier that prevents damage to the intestinal epithelial cells. When the trophozoites overcome this first barrier, the epithelial cells secrete potent pro-inflammatory mediators and chemokines that recruit immune cells, such as neutrophils, which release reactive oxygen species [15,16] and activated macrophages that release nitric oxide [16,17], both damaging the plasma membrane by lipid oxidation. Another protection mechanism of the intestinal epithelial cells is the production and secretion of antimicrobial peptides, such as LL-37 [18] and defensin 2 [19], which has been showed to damage the trophozoites *in vitro*. These peptides destabilize and alter the PM of *E*. *histolytica*, causing an increase in its permeability, reducing the viability of the amoebae. The human complement system is one of the most effective strategies to prevent the dissemination of trophozoites, once the amoebae activate the complement system, the membrane attack complex is formed which lyses the trophozoites [20,21].

In response to the attack of the human immune system, the amoeba confronts it and neutralize the potential damage through molecules called virulence factors. The most relevant molecules are: *i)* Gal/GalNAc lectin that binds to colonic mucin and promotes adhesion to the host cell [22,23] and linking to C8 and C9 subunits interrupting the assembly of the complement on the membrane of the trophozoites [24]; *ii)* cysteine proteases that degrade antibodies such as IgA and IgG [25,26] and the C3 convertase involved in the activation of the classic complement pathway and the amplification of the inflammatory process [21]; also, these enzymes degrade IL-1β, reducing the production of reactive oxygen species and nitric oxide by neutrophils and activated macrophages [27–29]; *iii)* amoebapores, which are small pore-forming proteins that have the ability to lyse eukaryotic cells in a cell contact-dependent manner [30–32]. Also, there is another set of molecules called virulence determinants that are indirectly involved in the pathogenic process by regulating the expression of virulence factors, with high gene expression plasticity and conferring adaptation and survival of the amoeba [33]. *E*. *histolytica* is an infectious agent that during the invasive process, either in the intestine or the liver, confronts the different defenses of the host, such as inflammatory response and the complement or the antimicrobial peptides that can directly damage its PM, requiring a mechanism for membrane repair and prevent its cell lysis.

In the present work, we evaluated the participation of aSMases in the PM damage repair in *E*. *histolytica*. The analysis of the *E*. *histolytica* genome revealed the presence of six genes encoding for aSMases that are transcribed under basal growth conditions. The *EhaSM6* coding sequence generates a functional recombinant protein with aSMase activity. The aSMase activity secreted by the virulent strain HM-1:IMSS is stimulated by Mg^+2^ and inhibited by Co^+2^, similarly as for the recombinant enzyme. The *EhaSM6* overexpression in *E*. *histolytica* trophozoites induces an increase in the secreted activity and tolerance to lysis with pore-forming molecules (β-Defensin 2, human complement, Streptolysin O and Magainin), in a Ca^2+^-dependent process. This PM damage induced an increase in the expression of *EhaSM1*, *2*, *5* and *6* genes, meanwhile oxidative stress induced an increase in all of them. Furthermore, we found that the damage to the PM of *E*. *histolytica* induces the exocytosis of lysosomal aSMase, resulting in the formation of membrane patches and endosomes. We propose that membrane damage promotes the migration of lysosomes towards the exposed site of the lesion where they secrete aSMase that induce the endosome formation and thus internalize the lesion, regenerating the integrity of the PM, favoring the amoebic viability and survival. We report for the first time that *E*. *histolytica* possess a mechanism of PM damage repair mediated by aSMase.

## Results

### Genes encoding acid sphingomyelinases in *E*. *histolytica* are actively transcribed

**Fig 1:**
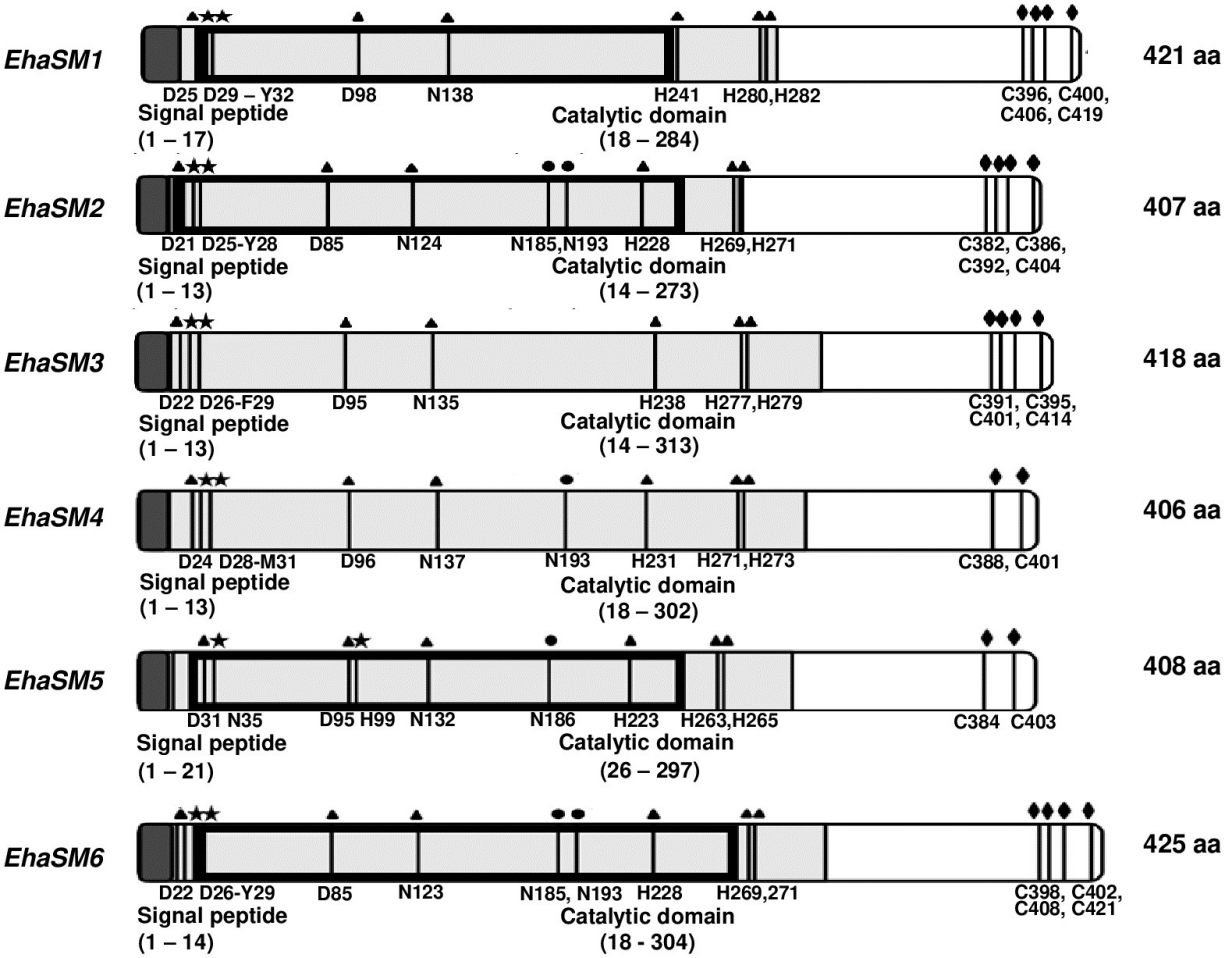
Schematic illustration of the functional motifs presents in the *Entamoeba histolytica* aSMases. aSMases in *E*. *histolytica* are small proteins (406–425 aa) containing a signal peptide in the N-terminal region (dark gray) followed by a metallophosphoesterase catalytic domain (light gray) and a calcineurin domain only for *EhaSM* 1,2,5 and 6 (black box). In addition, triangles (▲) show predicted metal coordinating residues, circles (●) represent conserved residues involved in substrate recognition loop motif “NX3CX3N”. The residues included in the hydrophilic/aromatic cluster are marked with stars (**⋆**). C-terminal cysteines are shown with rhombi (**◆**).

A search for annotated sequences encoding aSMases was done in the *E*. *histolytica* genome database [34]. Six genes annotated as putative aSMases-like phosphodiesterases were found: *EhaSM1* (EHI_040600), *EhaSM2* (EHI_172510), *EhaSM3* (EHI_118110), *EhaSM4* (EHI_100080), *EhaSM5* (EHI_147020) and *EhaSM6* (EHI_125660). The analysis of the six identified coding sequences for aSMases revealed that they present homology at the protein sequence level. The identified sequences showed conserved domains, but overall, the homology ranged between 11 to 50%, with identity ranging between 11 to 50% (S1 Fig). The alignment of the predicted amoebic sequences with those corresponding to aSMases in other organisms (*Homo sapiens* and *Mus musculus*) (S1 Fig) showed that the all the amoebic sequences exhibited the amino acids involved in catalysis, that have been described for these enzymes, which includes conserved amino acids important for catalysis, sites for metal coordination, the hydrophilic-aromatic cluster, the substrate recognition site "NX3CX3N" [35], the cysteines involved in the processing of the C-terminal region associated with the activation of the enzyme [36,37], and N-terminal signal peptide that suggests these enzymes are secreted [38] (Fig 1). These conserved residues match with those reported for the aSMases of the nematode *Caenorhabditis elegans*, which has a 30% homology with the human aSMase [39]. The *in silico* analysis of amoebic aSMases revealed that coding sequence renders proteins ranging between 46–49 kDa with a predicted signal peptide of 14 to 20 residues. Also, no transmembrane regions or signals for endoplasmic reticulum retention were identified, which suggests that these proteins are secreted. In addition, four aSMases have a calcineurin-like region (*EhaSM1*, *EhaSM2*, *EhaSM5*, and *EhaSM6*) (Fig 1), which suggests their participation in response to stress conditions [40]. qRT-PCR analysis showed that all the putative aSMase genes are transcribed and the *EhaSM6* showed the highest expression (60%) under basal growth conditions (S2 Table).

### Effect of divalent cations and kinetics of secretion of aSMase activity

**Fig 2:**
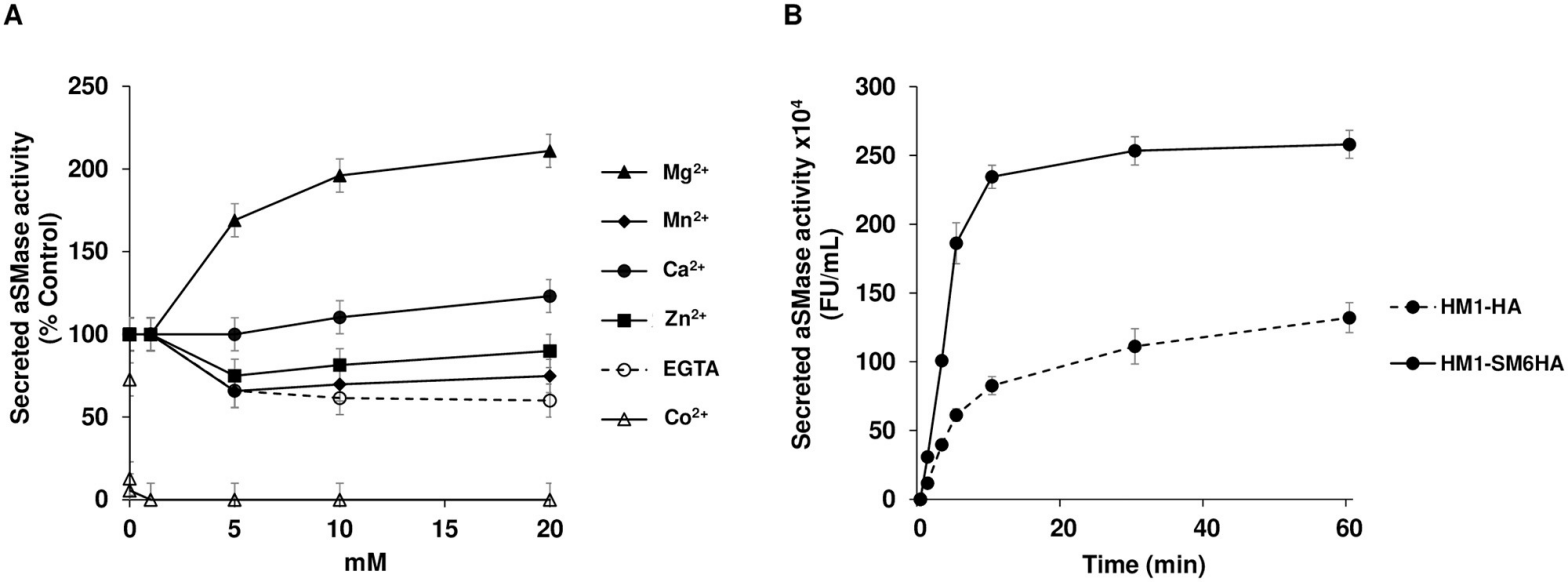
Secreted aSMase activity in *E*. *histolytica*. **A**. Effect of divalent cations on secreted aSMase activity. After 10 min of incubation, the secreted aSMase activity was determined in the presence of different concentrations of Mg^2+^, Mn^2+^, Ca^2+^, Co^2+^, Zn^2+^ or EGTA (0–20 mM). The percentage of activity of aSMase was determined by comparing with the basal activity in the absence of divalent cations. **B**. Kinetics of the secretion of aSMase activity. The secreted activity of aSMase in DMEM medium at different times of incubation at 37°C. Continuous line, secreted aSMase activity of HM1-SM6HA strain; discontinuous line, the secreted aSMase activity of HM1-HA strain. The results show the average of three independent experiments in duplicate.

We found that the six predicted amoebic aSMases genes are transcribed in the HM1 strain under basal growth conditions. Although these putative aSMase enzymes share sequence similarity with well-characterized mammalian aSMases, it is not known if these genes code functional aSMases proteins. The expression analysis showed that *EhaSM6* exhibited the highest expression, for this reason, we cloned the complete ORF in a suitable vector and the recombinant protein was produced in *E*. *coli*. The purified rEhaSM6 exhibited aSMase activity stimulated by Mg^2+^ and inhibited by Co^2+^ (S3 Table). In contrast with previous studies showing that the mammalian enzyme was stimulated by Zn^2+^ [41,42], the amoebic rEhaSM6 was not significantly affected by Zn^2+^ (S3 Table). The aSMases of *E*. *histolytica* have a predicted signal peptide, therefore, the secreted aSMase activity was determined from trophozoites in the exponential growth phase. After 10 min incubation, the secreted activity of HM-1:IMSS trophozoites was determined in the presence of different concentrations (0–20 mM) of divalent cations (Mg^2+^, Mn^2+^, Ca^2+^, Co^2+^ or Zn^2+^), likewise, the EGTA chelator was used to scavenge for trace amounts of metal ions. Mg^2+^ at a concentration of 20 mM stimulated the activity 2.1-fold, while Ca^2+^ and Zn^2+^ did not show a significant effect. Mn^2+^ and EGTA at a concentration of 20 mM inhibited the activity in approximately 40%, while Co^2+^ totally inhibited the activity at concentrations even lower than 1 mM (Fig 2A), presenting the same cation requirements or inhibitory effect as for the rEhaSM6.

To evaluate the biological role of aSMases in the amoebic trophozoites, the aSMase6 was further studied by generating a construct to overexpress it. Transfectant trophozoites overexpressing the *EhaSM6* gene fused to an HA tag in the C-terminus were obtained. The transcript level of *EhaSM6* was evaluated in the overexpressing strain (HM1-SM6HA) by qRT-PCR. The transcript level was 7.2-fold higher in comparison with the control strain transfected with the empty plasmid (HM1-HA) (Table 1). We measured the secretion of aSMase activity in the control and overexpressing strains at different times of trophozoite incubation. The results indicate that the activity of secreted aSMase in the overexpressing strain increases 3-fold in the first 10 min of incubation in comparison with the control strain. After 30 min the secreted activity remains constant (Fig 2B). Secreted aSMase activity was not detected in trophozoites incubated at 4 °C.

**Table 1:** Quantitative expression levels of *EhaSM* genes of *E*. *histolytica* in response to SLO exposition.

Strain	HM1-HA	HM1-HA + SLO	HM1-SM6HA	HM1-SM6HA + SLO
*EhaSM genes*				
***EhaSM1***	1.0	3.98 ± 0.57	1.14 ± 0.55	4.66 ± 0.44
***EhaSM2***	1.0	2.95 ± 0.78	1.75 ± 0.22	2.58 ± 0.96
***EhaSM3***	1.0	1.43 ± 0.51	1.46 ± 0.35	1.60 ± 0.35
***EhaSM4***	1.0	1.17 ± 0.21	1.33 ± 0.15	1.35 ± 0.11
***EhaSM5***	1.0	2.56 ± 0.37	0.96 ± 0.29	2.89 ± 0.49
***EhaSM6***	1.0	4.16 ± 0.93	7.23 ± 0.82	9.49 ± 0.37

Data were normalized using the ΔΔCT method against the housekeeping gene *Ehgapdh*. The control HM1-HA strain expression ratio is set to 1.0 and that the values represent the fold of over-expression. * Trophozoites treated with 1.6 ng/μL of SLO for 3 min at 37 °C.

### Damage to the amoebic plasma membrane induces the secretion of aSMase activity and increases cell viability

**Fig 3:**
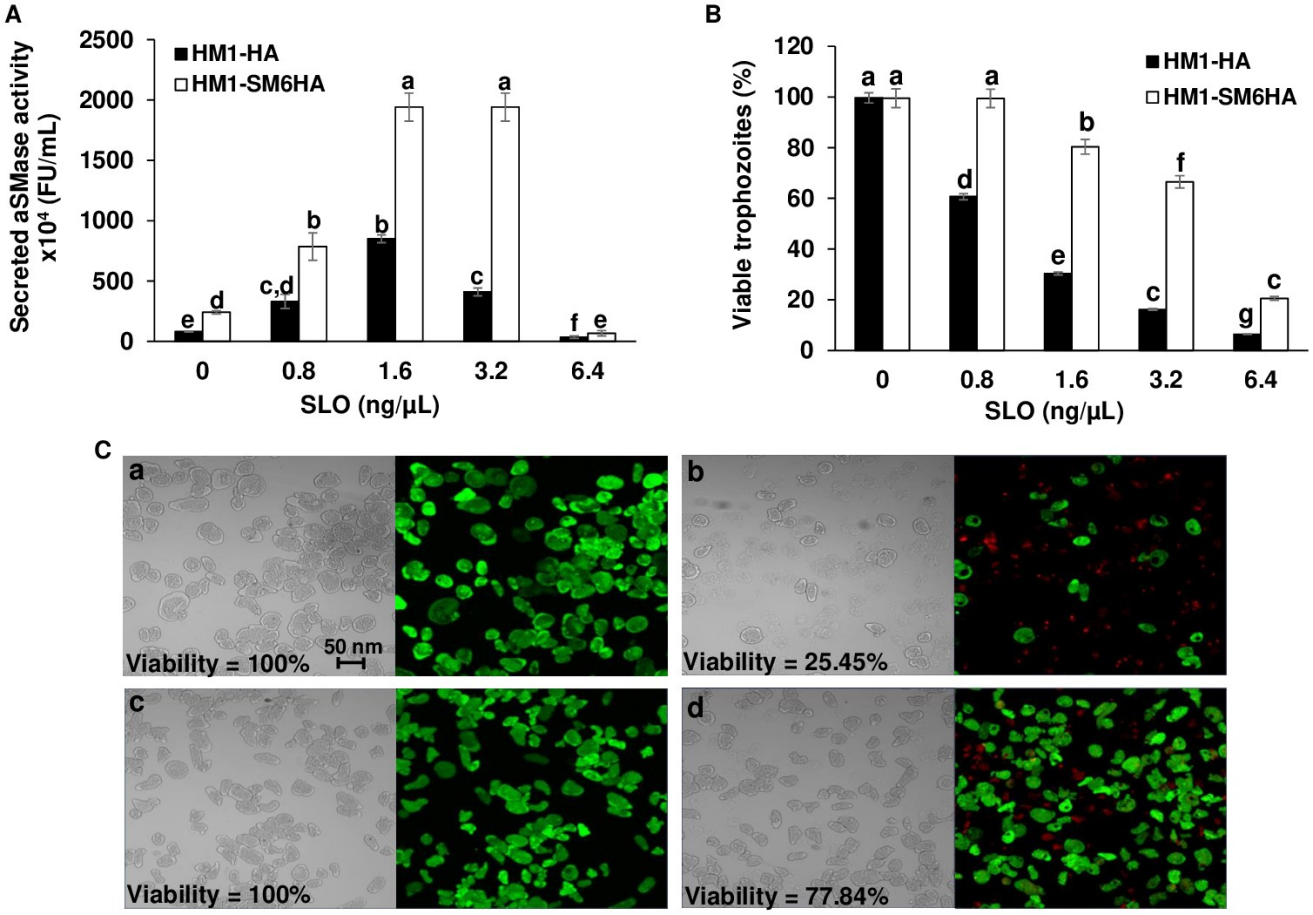
Secreted aSMase activity and viability of trophozoites treated with SLO. **A**. aSMase activity detected in cell-free supernatants of HM1-HA and HM1-SM6HA strains, collected after three min at 37 °C in DMEM medium with different concentrations of SLO (0.8–3.2 ng/μL). **B**. The viability of trophozoites treated with SLO (0.8–6.4 ng/μL) exposed to the toxin for 3 min. The percentage (%) indicates the viability of trophozoites by the exclusion of trypan blue. Different letters over the bars represent statistically significant differences at P ≤ 0.05 (Tukey–Kramer test). **C**. The viability of the trophozoites was also determined using the Live/Dead staining. Live cells were measured by enzymatic conversion of permeable calcein-AM to fluorescent calcein (green). The dead cells were detected by the absorption of ethidium homodimer in the nuclear DNA (red). The percentage (%) indicates the viability of the trophozoites by counting the green stained cells from a total of 250 cells. a. HM1-HA strain and b. HM1-SM6HA strain without exposing to SLO. c. HM1-HA strain and d. HM1-SM6HA strain exposed to SLO (1.6 ng/μL) for three min in DMEM medium containing 1.8 mM Ca^+2^.

SLO is an exotoxin from group A streptococci, which is toxic to eukaryotic cells due to its affinity for cholesterol, but at controlled doses promotes the formation of membrane pores that in consequence triggers the secretion of aSMase required for membrane repair in mammalian cells [43,44]. The effect of SLO on the secretion of aSMase activity in trophozoites of HM1-HA and HM1-SM6HA strains was evaluated. Different concentrations of toxin were tested (0.8–6.4 ng/μL), and the secreted activity of aSMase in trophozoites of amoebic strains was determined. Both strains showed an increase in the secreted activity, reaching a maximum of 8-fold with 1.6 ng/μL of SLO (Fig 3A). The secreted aSMase activity in the HM1-SM6HA is at least 2-fold higher than for HM1-HA in all the SLO treatment conditions, except with 6.4 ng/μL where there is a decrease in activity for both strains with respect to toxin-free amoebas. Also, after SLO treatment the amoebic viability was analyzed. The control HM1-HA strain showed a gradual decrease in cell viability, reaching 15% of viability at 3.2 ng/μL of SLO, this is in contrast with the 65% of viability for the *EhaSM6* overexpressing strain (Fig 3B). Interestingly, 6.4 ng/μL of SLO, a lytic concentration of the toxin, is associated with the lowest levels of enzyme secretion, suggesting that the secreted activity undergoes an activation process. Therefore, a greater increase in the secreted activity of aSMase is related to enhanced amoebic viability after SLO treatment.

The viability of trophozoites of both strains was also evaluated using Live/Dead staining. After treatment with 1.6 ng/μL of SLO for three min, the viability of HM1-HA and HM1-SM6HA strains were 25.45% and 77.84%, respectively (Fig 3C). The viability values were similar to those obtained by the exclusion of trypan blue (Fig 3B).

To rule out that the rise in aSMase activity in the supernantat after SLO tratment, alcochol dehydrogenase (ADH), a cytoplasmic enzyme, activity was measured in *E*. *histolytica* trophozoites. Activities of aSMase and ADH were monitored over time to determine a correlation to cellular integrity. By measuring the release of the cytoplasmic marker enzyme ADH in trophozoites of both strains, only 15% of total activity was released from amoebae without and with low SLO exposition (1.6 ng/μL). While the secreted aSMase activity was present in amoebic supernatants without SLO treatment and a significant increase of the secreted activity (>8-fold) was observed when amoebae were exposed to the same low concentrations of SLO. In contrast, lytic SLO concentration (6.4 ng/μL) released a substantial ADH activity in comparison with the very low aSMase activity detected for both amoebic strains. It became apparent that the release of ADH was accompanied by cellular disintegration caused by high SLO concentration (6.4 ng/μL). Using the tool of ADH release as a criterion for cellular disintegration, the majority of amoebae appeared to be intact in DMEM medium (S2 Fig). At least in this condition, the majority of aSMase activity has been released by viable amoebae. With regard to aSMases, the increased activity detected in the supernatants were found to depend on the SLO concentration and amoebic viability, indicating that the aSMase is secreted and activated only upon plasma membrane damage response in viable trophozoites with active secretory machinery.

### Amoebic viability after toxin exposition is related to the secreted aSMase activity secretion in a Ca^2+^ dependent process

**Fig 4:**
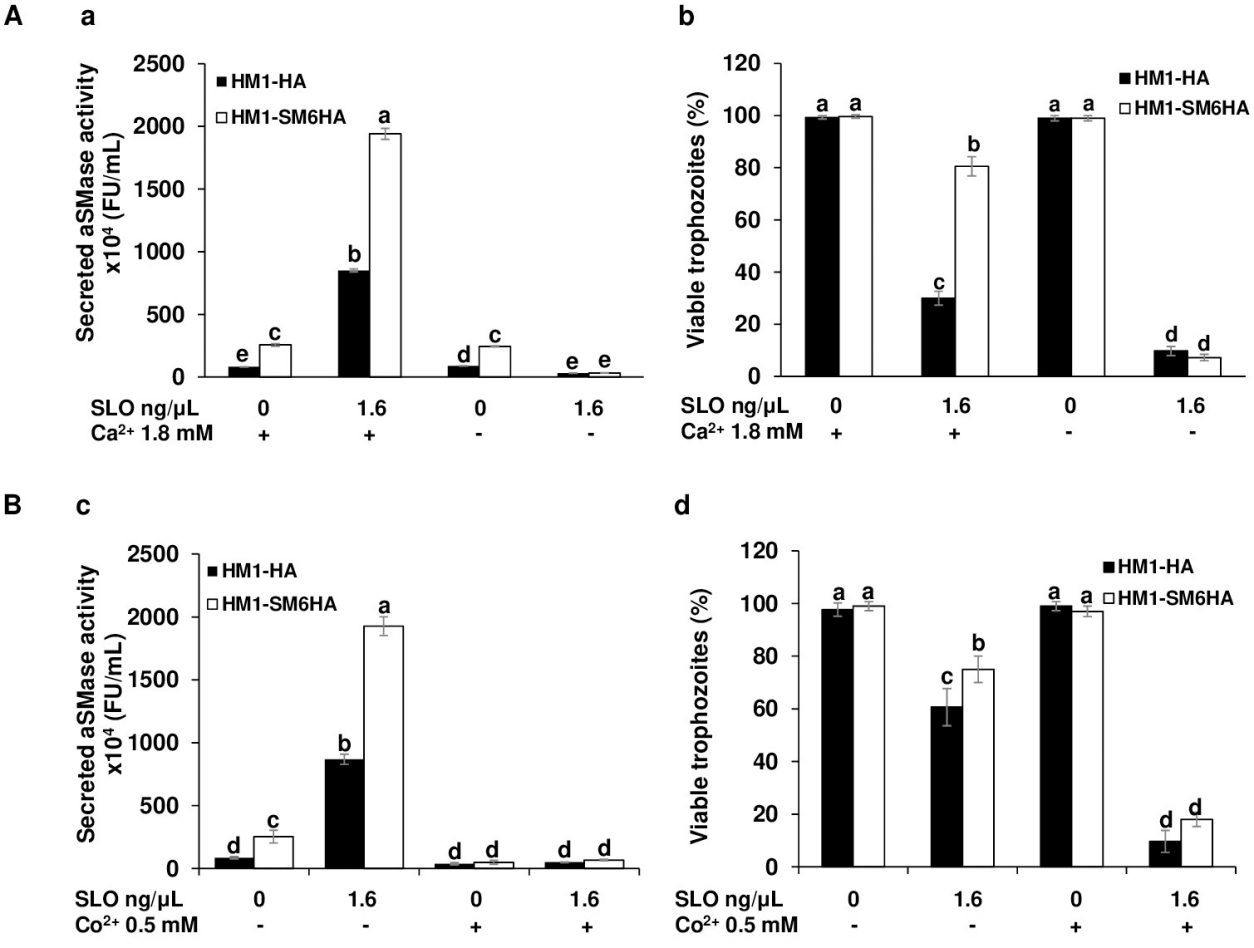
Secreted aSMase and viability of *E*. *histolytica* trophozoites treated with SLO are a Ca^2+^ and enzyme activity dependent processes. **A**. Calcium dependence. a. Secreted aSMase activity after three min of exposition to SLO (1.6 ng/μL) in complete DMEM medium and Ca^2+^-free DMEM in HM1-HA and HM1-SM6HA strains. b. The viability of trophozoites treated with SLO (1.6 ng/μL) for three min with or without Ca^+2^. **B**. Inhibition of aSMase activity. c. Secreted aSMase activity after three min of exposition to SLO (1.6 ng/μL) in complete DMEM medium and 0.5 mM of Co^2+^ in HM1-HA and HM1-SM6HA strains. d. The viability of trophozoites treated with SLO (1.6 ng/μL) for three min or without Co^2+^. The percentage (%) indicates the viability of the trophozoites by the exclusion of trypan blue. Different letters over the bars represent statistically significant differences at P ≤ 0.05 (Tukey–Kramer test).

The Ca^2+^ gradient that infiltrates through cell membrane lesions activates cytoplasmic proteins and induce the exocytosis of lysosomes at the site of the lesion [45]. Lysosomes release their cargo, including the aSMase, followed by massive endocytosis that triggers the process of wound repair [8,44,46]. The secreted aSMase activity and viability of amoebic trophozoites were evaluated in the presence of 1.6 ng/μL of SLO but using a Ca^2+^-free DMEM medium. No increase of secreted aSMase activity was observed in trophozoites of HM1-HA and HM1-SM6HA strains treated with SLO in the absence of extracellular Ca^2+^; in contrast with the increase in enzymatic activity in the presence of extracellular Ca^2+^. The secreted activity of aSMase in the absence of toxin was not affected by the presence or absence of Ca^2+^ in both strains (Fig 4Aa). The results show that after inducing damage with SLO in the presence of 1.8 mM of Ca^2+^ for three min, the viability of the control strain is 30%, while that of the *EhaSM6* over-expressing strain is 80%. In contrast, in the absence of Ca^2+^ and damage with SLO, the viability decrease was more evident, since the viability for both strains is less than 11% in comparison to their controls in the absence of damage under the same conditions (Fig 4Ab). Therefore, Ca^2+^ is a necessary cation for exocytosis of lysosomes and the secretion of aSMase activity, which correlates with the preservation of amoebic viability as it has been demonstrated in other cell types that trigger the repair of damage to the PM [47].

As it has been shown above (Fig 2), the aSMase activity from *E*. *histolytica* was strongly inhibited by Co^2+^. We perform an assay of plasma membrane repair after SLO exposition in the presence of Co^2+^ 0.5 mM. The Fig 4Bc shows that after the exposition to the SLO, the secreted aSMase activity was inhibited by Co^2+^, which also inhibited the membrane repair capacity of the amoebae and consequently decreased their viability (Fig 4Bd). This strongly suggests that aSMase6 is one of the enzymes that initiate the repair mechanism of damage to the PM of the amoeba.

### Effect of the β-Defensin 2 and human complement on the secretion of aSMase activity and viability of *E*. *histolytica*

To evaluate if the aSMase6 is involved in the repair of membrane damage generated by another membrane damaging agent, we used the antimicrobial peptide β-Defensin 2. During an intestinal human infection, several antimicrobial molecules are produced, where β-defensins are the most common [48]. Defensins are cysteine-rich cationic peptides of low molecular weight, 3 to 5 kDa, that bind to the membranes of microorganisms rich in anionic phospholipids [49] and integrate into the membranes inducing the formation of pores [50]. In this study, the HM1-HA and HM1-SM6HA strains were exposed to increasing concentrations of β-Defensin 2.

**Fig 5:**
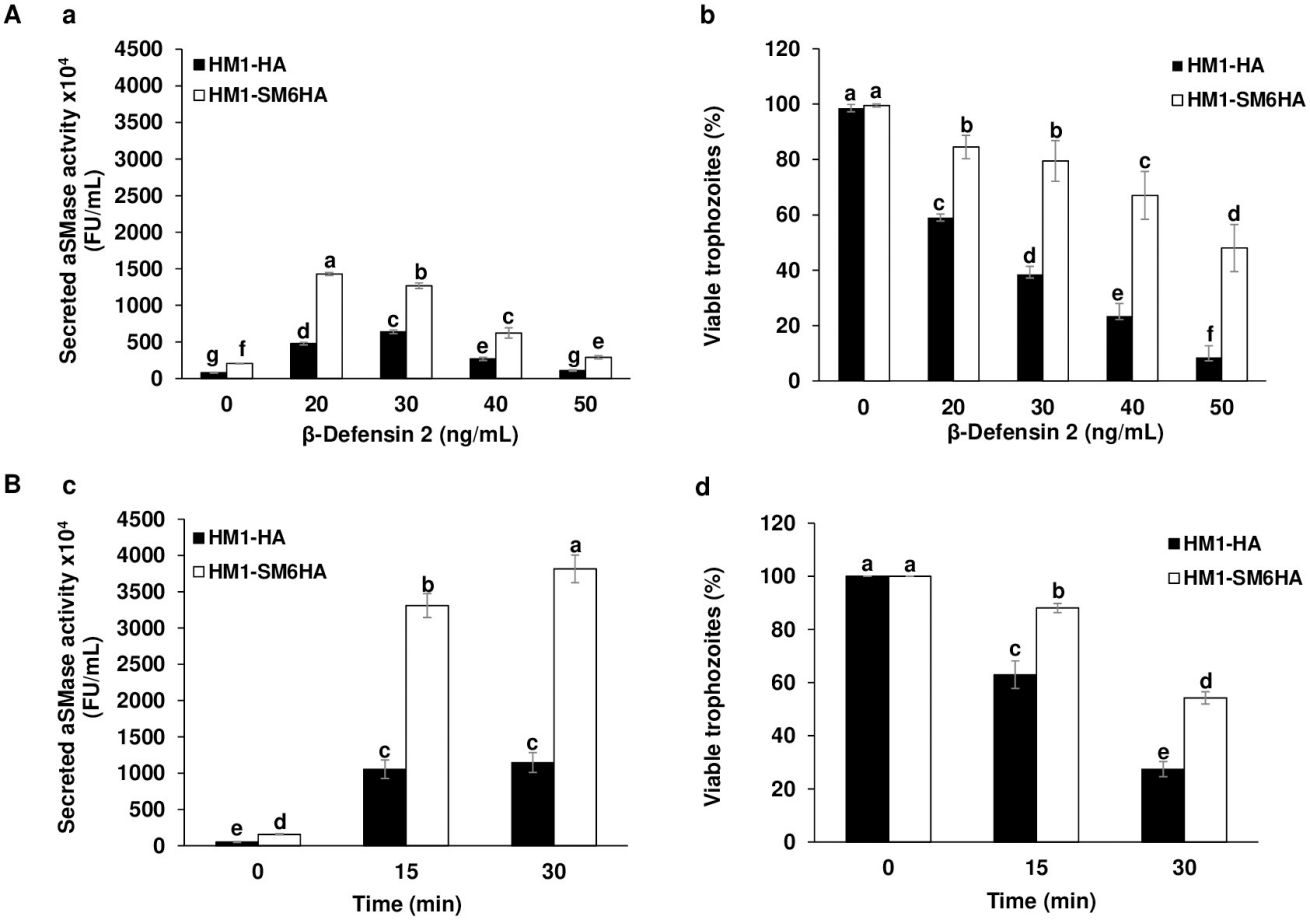
Effect of β-Defensin 2 and human complement on the secreted aSMase activity and viability of *E*. *histolytica* trophozoites. **A**. Trophozoites treated with β-Defensin 2. a. Secreted aSMase activity after 30 min of exposition to β-Defensin 2 in complete DMEM medium with and Ca^2+^ in HM1-HA and HM1-SM6HA strains. b. Viability of trophozoites after interaction with β-Defensin 2. **B**. Trophozoites in interaction with human complement. c. Secreted aSMase activity after 0, 15 and 30 min of exposition to human complement in complete DMEM medium with and Ca^2+^ in HM1-HA and HM1-SM6HA strains. d. Viability of trophozoites after interaction with human complement. The percentage (%) indicates the viability of the trophozoites by the exclusion of trypan blue. Different letters over the bars represent statistically significant differences at P ≤ 0.05 (Tukey–Kramer test).

Concentrations of 20–30 ng/mL of β-Defensin 2 induced the highest secretion levels of aSMase activity reaching 6-fold and 7-fold for the control and overexpressing strains respectively, which is 2-fold higher in the overexpressant than the control strain (Fig 5Aa). Higher levels of secreted activity correlate with higher capability of PM repair and consequently greater viability (Fig 5Ab). At higher concentrations of β-Defensin 2, the levels of secreted activity decreases, as does the viability of trophozoites, again suggesting that the secretion of aSMases is a process that requires active cellular metabolism.

The human complement system is an effective defense mechanism in the elimination of pathogens, which culminates in the formation of large pores in the membrane, which alters the osmotic equilibrium of the pathogen [51,52]. The effect of the complement on the secreted aSMase activity and viability of the trophozoites of HM1-HA and HM1-SM6HA strains was evaluated. The results obtained indicate that after incubation with the complement, both strains significantly increase the activity of secreted aSMase, showing a 3-fold increase in the overexpressant with respect to the control strain (Fig 5Bc). Furthermore, viability results (Fig 5Bd) show that amoebae interacting with complement diminish their viability, but this decrease was more evident for the control strain than for the overexpressing strain, which bears a more efficient repair mechanism. Complement exposition induced the highest levels of secreted aSMase activity in *E*. *histolytica* than all the pore-forming peptides evaluated in this work.

Another plasma membrane disrupting agents were evaluated such as Magainin, an antimicrobial peptide and, Triton X-100, an non-ionic detergent. Magainin is a cathelicidin that interacts with the plasma membrane forming permeable ion channels [47,48]. Trophozoites exposed to increasing concentrations of Magainin exhibit similar results to those obtained with the other pore-forming molecules evaluated in this work, where the higher secreted aSMase activity of the HM1-SM6HA correlated with the higher amoebic viability (S3A Fig). When Triton X-100 was used at concentrations that acts as a permeabilizer [53], a slight increase in the secreted activity of aSMase was observed, but at higher concentrations, it produces lysis of the amoebae and very low levels of activity was detected (S5B Fig).

### Effect of hydrogen peroxide on aSMase activity and viability of trophozoites of *E*. *histolytica*

**Fig 6:**
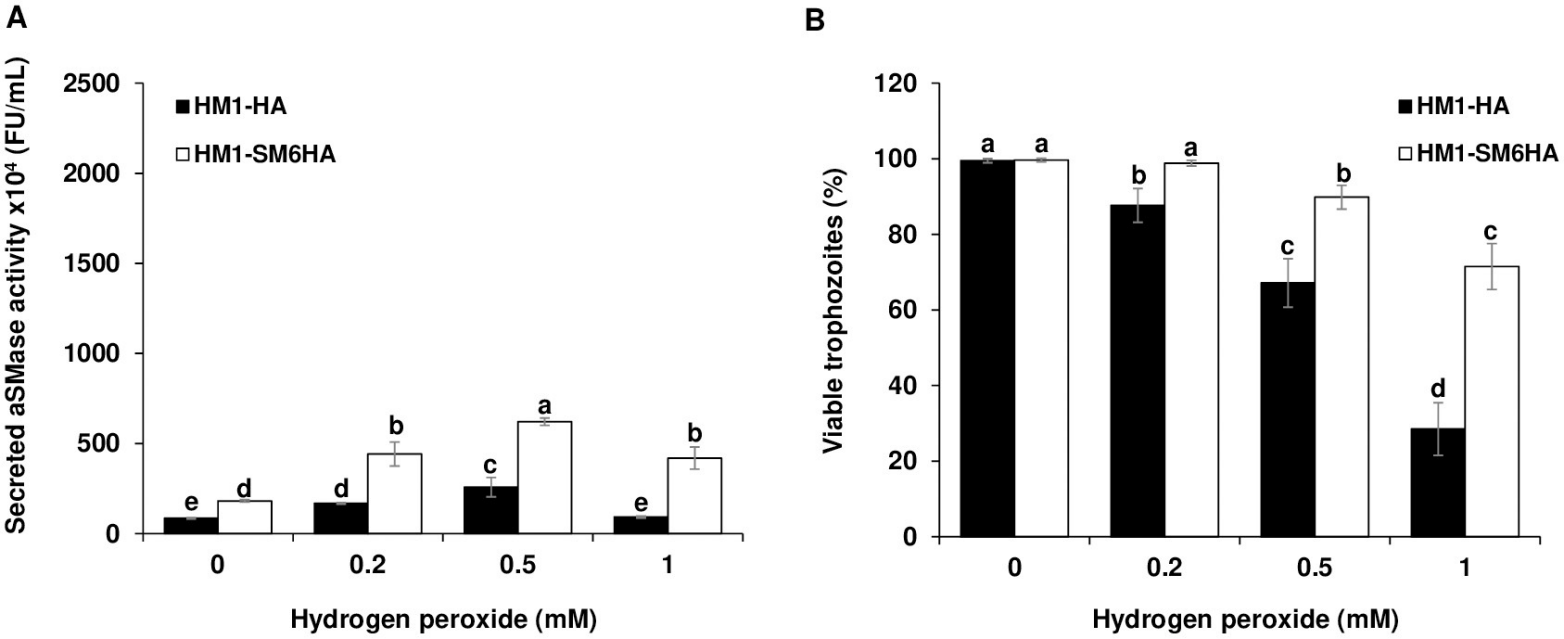
Effect of hydrogen peroxide on the secreted aSMase activity. **A**. Secreted aSMase activity by trophozoites exposed to different concentrations of H_2_O_2_ (0.2–1 mM) in DMEM medium for 10 min. **B**. Viability of trophozoites treated with different concentrations of H_2_O_2_. The percentage (%) indicates the viability of the trophozoites by excluding trypan blue. Different letters over the bars represent statistically significant differences at P ≤ 0.05 (Tukey–Kramer test).

It has been reported that the generation of ceramide, mainly a product of the hydrolysis of sphingomyelin by SMases, is associated with different stimuli such as oxidative stress generated by H_2_O_2_, generating cellular responses such as apoptosis and cell proliferation [54]. To determine the possible role of aSMases in response to damage by oxidative stress in *E*. *histolytica*, the effect on the secreted activity of aSMases and cell viability in the HM1-HA and HM1-SM6HA strains was determined. Trophozoites of HM1-SM6HA strain showed a significant increase in the secreted activity of the aSMase in all peroxide concentrations evaluated with respect to that observed in the HM1-HA control strain (Fig 6A); these increase in the activity of aSMase is related to a maintenance of the viability (Fig 6B) in each of the peroxide concentrations evaluated in the HM1-SM6HA strain. However, the level of secreted aSMase activity is lower than those obtained with pore-forming molecules.

### Damaged trophozoites present exocytosis of lysosomes and increased endocytosis

**Fig 7:**
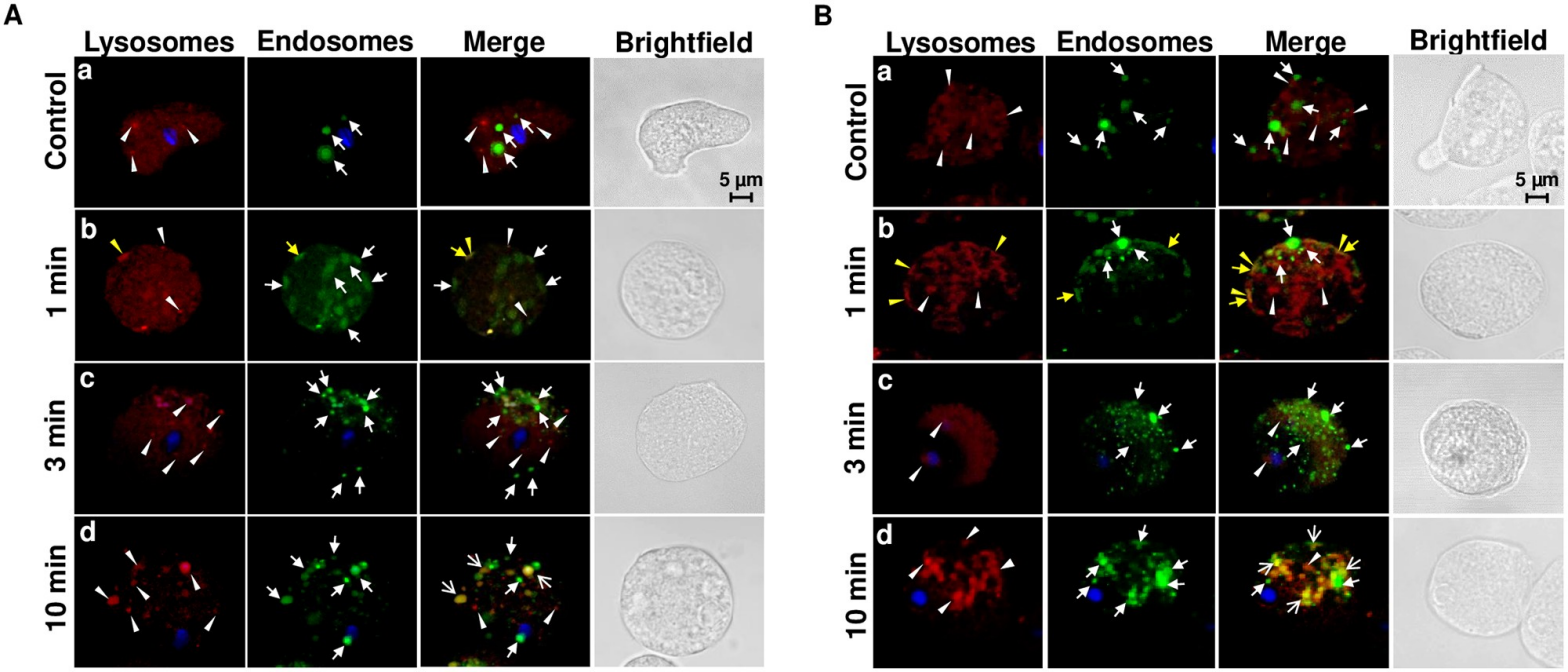
Plasma membrane damage induces lysosome exocytosis followed by endocytosis in *E*. *histolytica* trophozoites exposed to SLO. Trophozoites of HM1-HA (A) and HM1-SM6HA (B) strains incubated at 37 °C with Lysotracker (2 μM), FITC labeled-Dextran (0.25μg/μL), SLO (1.6 ng/μL) and nuclei were stained with Höechst 33342. Trophozoites without SLO exposition (a) and trophozoites exposed to SLO for one min (b), three min (c), and 10 min (d). Arrowheads show positive compartments with Lysotracker, closed arrows indicate endosomes, yellow arrows indicate the formation of patches and open arrows the endolysosomal compartment.

The above evidence strongly indicates that the aSMase6 of *E*. *histolytica* is involved in membrane repair. To evaluate the events related to PM damage repair induced by SLO in HM1-HA and HM1-SM6HA strains, trophozoites labeled with Lysotracker from both strains were incubated at 37 °C with FITC labeled-Dextran and 1.6 ng/μL of SLO for short times. The HM1-HA control and HM1-SM6HA overexpression strains without exposing to SLO show the presence of lysosomes (red) and few endosomes (green) in the cytoplasm of the amoebic trophozoites (Fig 7A and 7B). HM1-HA trophozoites treated with SLO (1.6 ng/μL) change their classic amoebic shape to a rounded shape after 1 min of incubation, and the migration of lysosomes to the PM is observed, some of them seems fused to PM forming elongated structures (patch-like), this resembles the structures formed in some damaged eukaryotic cells. In addition, the number of endosomes increases and some of them are also located in the plasma membrane, where few of them co-localize with lysosomes (yellow) forming structures with a patch-like shape.

After three min of SLO exposure, the patches on the plasma membrane disappear and the number of endosomes increases in the subcortical region of the PM. After 10 min of SLO exposure, the polarization of the endosomes is lost, and vesicles exhibiting the label for both lysosomes and phagosomes are observed, suggesting the formation of endolysosomes (yellow) (Fig 7A). The mobilization of vesicles resembles the mechanism of membrane damage repair in HeLa cells, fibroblasts and lymphoblasts, after exposure to SLO, where the lysosomes fuse to the membrane and release the aSMase which promotes the injury site endocytosis [8,44,46,47].

Transfectant trophozoites that overexpress aSMase6 (HM1-SM6HA strain) exposed to SLO under the same conditions described above, after one min of exposition, trophozoites showed a greater number of patches showing colocalization of the lysosome and endosome markers. After three min of SLO treatment, the patches are no longer present, and the endosomes are observed in the inner side of the plasma membrane without co-localization with lysosomes. After 10 min, a large number of endosomes fused with lysosomes (endolysosomes) were observed. We theorize that the formation of these structures is a consequence of efficient endocytosis of the lesions in the PM (Fig 7B) in the HM1-SM6HA strain.

Endosomes per cell were quantified, showing an average of 2.8, 8.2, 5.2 and 29.8 endosomes per amoeba in strains HM1-HA, HM1-HA with SLO, HM1-SM6HA and HM1-SM6HA with SLO, respectively. Likewise, the amoebae of each of the strains from identical conditions as above were lysed and the FITC fluorescence emitted by the endosomes was quantified (S5 Table), which corroborates the data above. These results suggest that the aSMase6 overexpression induce an efficient repair mechanism evidenced by a significant increase in the viability of the trophozoites (Fig 5B), reinforcing the role of aSMase6 in PM repair.

**Fig 8:**
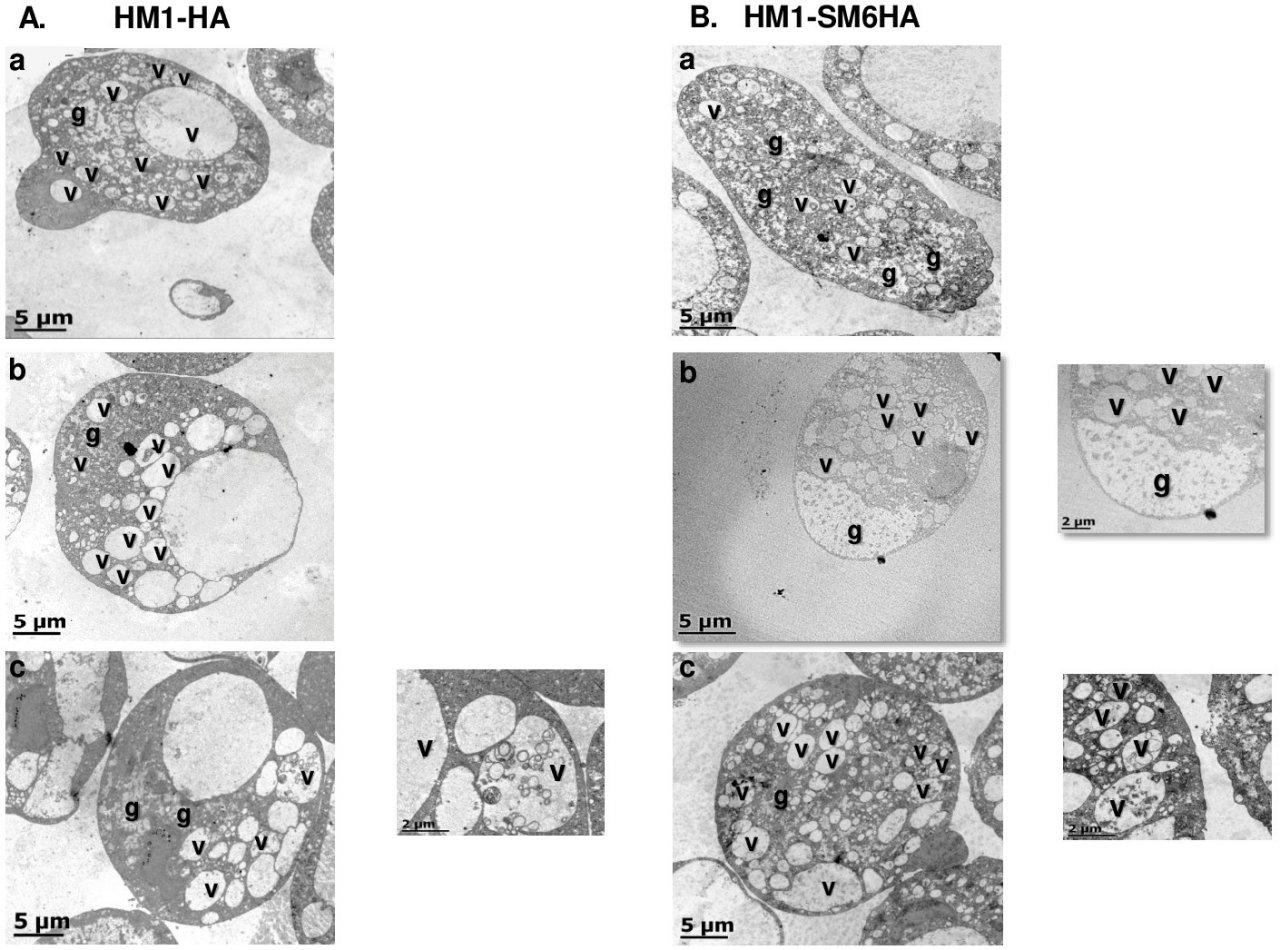
Transmission electron microscopy of *E*. *histolytica* trophozoites exposed to SLO. Trophozoites of HM1-HA (**A**) and HM1-SM6HA (**B**) strains. a, trophozoites without toxin; b, trophozoites exposed to SLO (1.6 ng/μL) for one min; c, trophozoites after three min of exposure to SLO. Vesicles (v) and glycogen (g).

With the above observations that revealed an intense mobilization of vesicles towards the plasma membrane, we sought the ultrastructural analysis of the trophozoites exposed to SLO by transmission electron microscopy (TEM). Trophozoites of HM1-HA and HM1-SM6HA strains, adhered to a plate, were treated with 1.6 ng/μL of SLO for one and three min, as described above. The trophozoites without SLO exposition, show a typical amoeboid morphology and cell structures, including several vesicles with little content inside (v) and glycogen (g) (Fig 8Aa and 8Ba). After one min of toxin exposure, the trophozoites of HM1-HA strain showed a polarization of vesicles towards the PM (Fig 8Ab), and after three min the vesicles presented residues of the PM (Fig 8Ac). Transfectant trophozoites that overexpress aSMase6 exposed for one min to SLO showed a stronger polarization of vesicles towards the site of membrane damage, displacing glycogen to the opposite end of the cell (Fig 8Bb). After three min, an increase of vesicles containing membrane residues was observed (Fig 8Bc).

The vesicles involved in membrane damage repair are mainly located polarized to one side of the PM because the SLO exposure was performed on adhered trophozoites, however when cells in suspension were used, the migration of vesicles was homogenously distributed around the PM and the glycogen was located on the center of the trophozoite (S4 Fig). In summary, fluorescence and transmission electron microscopy results strongly suggest that trophozoites of *E*. *histolytica* present a PM damage repair mechanism that renders viable trophozoites after PM damage.

### Endocytosis is induced by the recombinant protein aSMase6 and inhibited by Co^2+^ in trophozoites of *E*. *histolytica*

**Fig 9:**
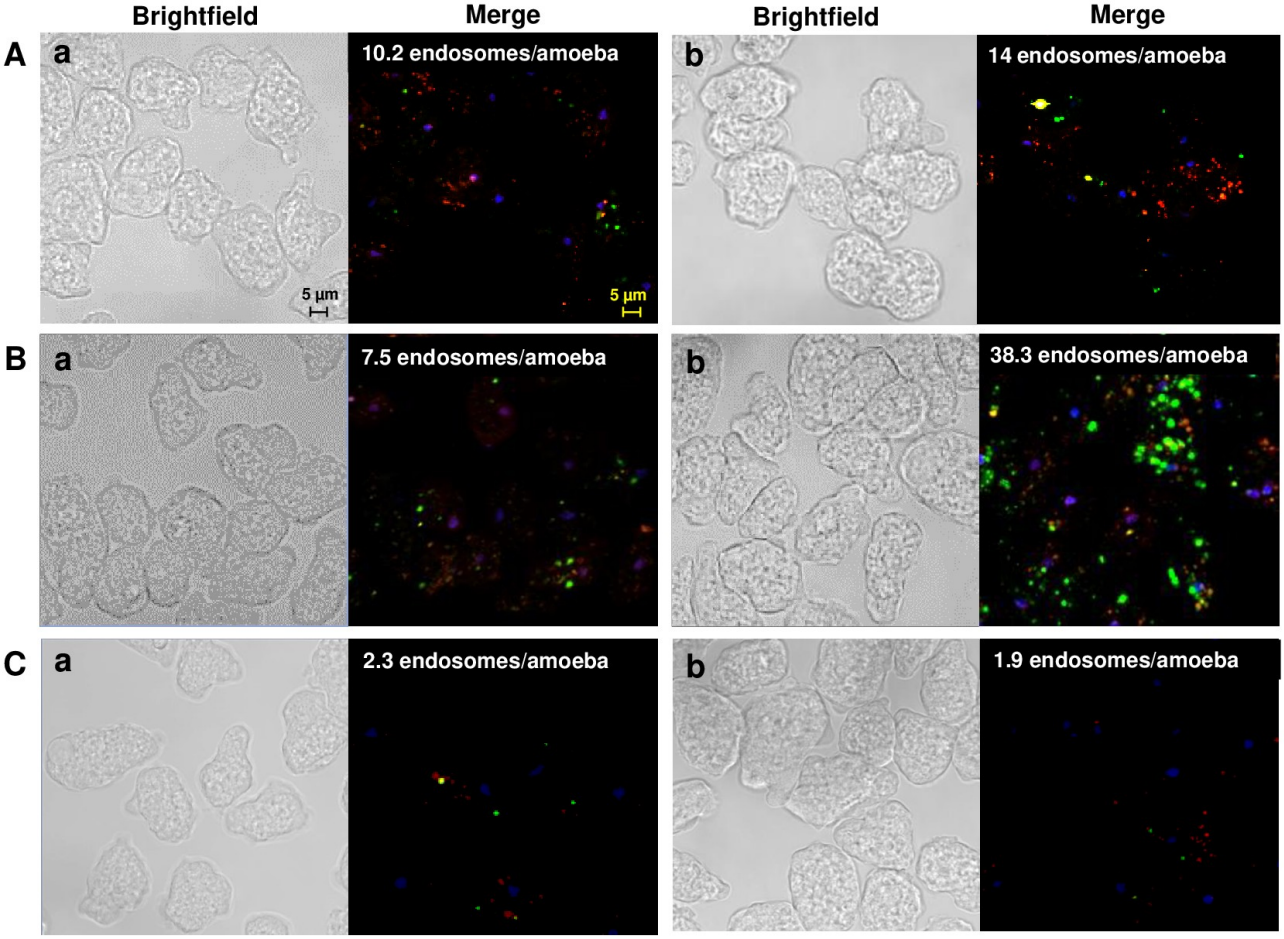
Formation of endosomes in *E*. *histolytica* trophozoites treated with the recombinant aSMase6 (rEhaSM6). Trophozoites of HM1-HA strain were treated with 20 μg of recombinant protein and FITC-Dextran (0.25 μg/μL) for one minute at pH 7.0 (A), pH 5.0 (B) plus Co^2+^ 0.5 mM (C). a, Trophozoites not treated with the rEhaSM6 protein. b, Exposed trophozoites to rEhaSM6. Nuclei were stained with Höechst 33342.

The results of confocal microscopy revealed that amoebae treated with toxin had an active process of endocytosis. To demonstrate that the secreted aSMase6 of *E*. *histolytica* participates in the repair mechanism by inducing the formation of endosomes, the amoebae of the HM1-HA strain were incubated with the protein rEHaSM6 and FITC -Dextran for 1 minute at neutral and acid pH. The results show that the trophozoites without the addition of the recombinant protein both at neutral pH (Fig 9Aa) and acid (Fig 9Ba), present an average of 10.2 and 7.5 endosomes per trophozoite, respectively. When the recombinant protein is added to the trophozoites for one minute at neutral pH in which the enzyme has no activity, the number of endosomes was similar to that of the control in this same condition (Fig 9Ab); meanwhile at acid pH an increase of 5-fold in the number of endosomes with respect to the untreated control was observed (Fig 9Bb). When Co^2+^ 0.5 mM was added to the HM1-HA strain at acidic pH, there was a decrease of endosome formation with respect to the amoebae without exposition to Co^2+^ (Fig 9Ca). In the presence of the recombinant protein and Co^2+^ 0.5 mM, the induction of endosome formation was not observed (Fig 9Cb). These results corroborate the participation of the aSMase6 in the formation of endosomes, which, are important structures for the internalization of the lesion in damaged PM.

### The release of cysteine proteases as a result of lysosome exocytosis

Our results suggest that amoebic trophozoites, in response to SLO exposition, fuse the lysosomes to the PM and thus releasing the aSMase along with its content into the extracellular milieu, generating a transient acidic environment necessary for aSMase activity as occurs in mammalian cells [44–46]. Cysteine proteases (CP) are present inside the lysosomes and thus should be released along with the cargo when lysosomes fuse with the PM during damage [55,56]. The CP activity secreted by HM1-HA and HM1-SM6HA strains was determined in the absence and presence of SLO. The HM1-HA and HM1-SM6HA strains exhibited similar levels of CP activity in the absence of damage, however, when the damage with the toxin is induced, this activity increases by 53% and 130%, respectively (S5 Fig), supporting the observation that the lysosomes indeed fuse with the PM releasing their cargo.

### N- and C- terminal processing of aSMase6 protein

**Fig 10:**
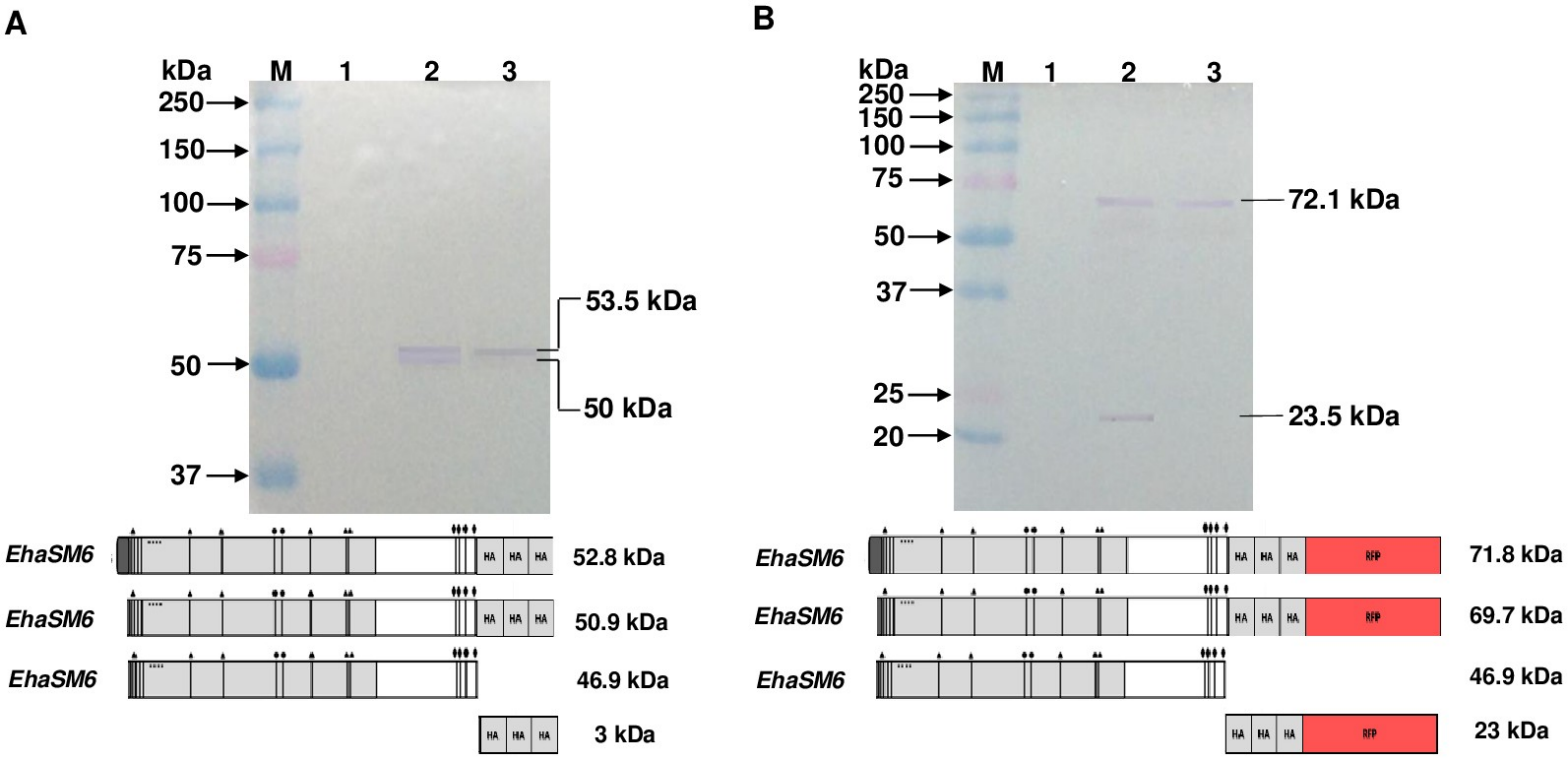
Immunodetection of aSMase6 in cellular fractions of transfectant trophozoites of *E*. *histolytica*. Trophozoites of HM1-SM6HA (**A**) and HM1-SM6HA-RFP (**B**) strains. Cell homogenates and concentrated supernatants were subjected to SDS-PAGE, transferred to nitrocellulose and immunodetected with monoclonal antibody anti-HA tag. Cell homogenate of the control strain (HM1-HA) (lane 1), cell homogenate of the corresponding strain (lane 2), and free-cell supernatant from the corresponding strain (lane 3).

*EhaSM6* gene code for a protein of 48.8 kDa with a predicted signal peptide indicating that the protein is secreted as reported for mammalian aSMases [57,58]. The cysteines located at the C-terminal suggests that the aSMase6 could be processed, as occur for the mammalian aSMase where one of the cysteines of this region is susceptible to proteolytic processing [36,59]. The HM1-SM6HA strain was used to detect the aSMase6 protein by Western blot using anti-HA antibodies. Despite the high levels of transcript in the over-expressing strain, two weak bands were revealed, suggesting that most of the protein was processed in its C-terminal end. Proteins of 53.5 and 50.9 kDa were detected in total homogenate, which corresponds to the predicted molecular weight of the unprocessed protein at the C-terminal region without and with N-terminal processing, respectively (Fig 10A, lane 2). A protein of 53.5 kDa was detected in the supernatant, which could correspond to the secreted aSMase6 unprocessed in the C-terminal or with post-translational modifications (Fig 10A, lane 3). The small differences found in the molecular weight of the proteins in contrast to the predicted requires further investigation of post-translational modifications of this enzyme.

To evaluate the C-terminal processing of the amoebic aSMase6, the HM1-SM6HA-RFP strain that produces the HA-tagged enzyme fused to a Red Fluorescent Protein (RFP) [60] in the C-terminal region was used. The activity of the enzyme was not disturbed by the addition of this tag since the supernatant activity is comparable to the HA fused protein described above (S6 Fig). Two proteins of 72 and 23.5 kDa were immunodetected with anti-HA antibodies in the total homogenate and one associated with the secreted protein with a molecular weight of 72 kDa (Fig 10B). In the HM1-SM6HA-RFP strain, the 72 kDa protein corresponds to the unprocessed protein, while the 23.5 kDa protein corresponds to the HA-RFP tag released from the C-terminal region of the aSM6HA-RFP protein (Fig 10B). These results indicate that there is a processing of the C-terminal end of the aSMase6. Analysis of the supernatant in the HM1-SM6HA-RFP strain revealed that only one protein of 72 kDa is detected corresponding to unprocessed aSM6HA-RFP protein, in contrast with the HM1-SM6HA strain where two bands were detected. If the N- and C-terminal unprocessed protein is present in the HM1-SM6HA-RFP strain, it is not distinguishable from the processed because the difference between these proteins is only of two kDa. The processed aSMase6 (N and C-terminal region) that would have a molecular weight of 47 kDa, in theory, should be more abundant than the remaining unprocessed protein that we observed in both strains. With the overall results, we demonstrate that the processing of aSM6 occurs at the N-terminal region to remove the signal peptide and the C-terminal region as described for the mammalian enzyme.

### Expression of the aSMase gene family of *E*. *histolytica* in response to damage to the plasma membrane

With the compiling results shown in this work, we suggest that the aSMase6 of *E*. *histolytica*, responds to the damage to the plasma membrane aiding to maintain cell viability. However, the amoeba has five additional aSMase genes (Fig 1), which until now their function remains unknown. The transcription levels of the *EhaSM1*, *EhaSM2*, *EhaSM3*, *EhaSM4* and *EhaSM5* genes besides the *EhaSM6*, were evaluated by qRT-PCR in the control (HM1-HA) and overexpressing (HM1-SM6HA) strains, before and after damage with SLO. Table 1 shows that the overexpressing strain has a 7.2-fold increase in the expression of the *EhaSM6* gene with respect to the parental strain, while the other 5 genes did not show a significant change in their expression. In response to PM damage with the SLO toxin, both strains showed an increase of the *EhaSM6* gene of 4.1 and 9.4-fold in the control and overexpressing strains, respectively. In response to SLO, there was an overexpression of 4, 3 and 2.5-fold of *EhaSM1*, *EhaSM2*, and *EhaSM5*, respectively. This suggests that, in addition to the aSMase6, the aSMase1, aSMase2, and aSMase5 could also be involved in the repair of membrane damage. In contrast, the genes that code for the aSMase3 and aSMase4 proteins did not show changes in their expression, suggesting they are not participating in the PM repair process or they exhibit a different regulation because of the absence of calcineurin region.

When evaluating the expression of the aSMase genes in *E*. *histolytica* trophozoites exposed to β-Defensin 2 30 ng/ml (S4 Table) or hydrogen peroxide 0.5 mM (Table 2), we found similar results with β-Defensin 2 to those obtained with amoebae exposed to SLO (Table 1) where the genes encode for aSMase3 and aSMase4 did not present changes in their expression. However, with amoebae exposed to hydrogen peroxide, all six aSMase genes showed a significative increase in the HM1-HA and HM1-SM6HA strains (Table 2), where the *EhaSM6* gene again presented the highest expression, even greater than that obtained with SLO and β-Defensin 2. Overall, these results suggest that aSMases 1,2,5,6 could be involved in the repair of plasma membrane damage caused by pore-forming molecules such as SLO and β-Defensin 2, while more widespread membrane damage, such as those caused by hydrogen peroxide, all aSMases could be involved.

**Table 2:** Quantitative expression levels of EhaSM genes of *E*. *histolytica* in response to hydrogen peroxide exposition.

Strain	HM1-HA	HM1-HA + Hydrogen Peroxide *	HM1-SM6HA	HM1-SM6HA + Hydrogen Peroxide*
*EhaSM genes*				
***EhaSM1***	1.0	3.24 ± 0.07	1.06 ± 0.17	3.73 ± 0.08
***EhaSM2***	1.0	5.04 ± 0.14	1.39 ± 0.08	5.02 ± 0.03
***EhaSM3***	1.0	3.14 ± 0.27	1.23 ± 0.15	3.27 ± 0.04
***EhaSM4***	1.0	6.48 ± 0.51	1.15 ± 0.09	6.51 ± 0.03
***EhaSM5***	1.0	6.09 ± 0.08	1.15 ± 0.09	6.08 ± 0.49
***EhaSM6***	1.0	9.22 ± 0.01	7.53 ± 0.21	18.86 ± 0.16

Data were normalized using the ΔΔCT method against the housekeeping gene *Ehgapdh*. The control HM1-HA strain expression ratio is set to 1.0 and that the values represent the fold of over-expression.

* Trophozoites treated with 0.5 mM of Hydrogen peroxide for 10 min at 37°C.

## Discussion

*Entamoeba histolytica* is able to invade human tissues by means of several molecules and biological properties related to virulence. Pathogenic amoebae use three major virulence factors, Gal/GalNAc lectin, amoebapore and proteases, to lyse, phagocytose, kill and destroy a variety of cells and tissues in the host [61–64], while its counterpart is the defensive response of the host that is characterized by humoral and cellular immune reactions [52,65,66]. The host-parasite relationship is based on a series of interplays between host defense mechanisms and parasite survival strategies.

In the present work, we characterized the aSMase activity and its role in PM repair. The genome of *E*. *histolytica* has six genes annotated as aSMases, which are actively transcribed and the *Ehasm6* gene has the most abundant transcript production rate. Amoebic aSMase sequences present low homology with other previously reported aSMases, such as the aSMases of *C*. *elegans* that shows 30% of homology with human and murine aSMases [39]. The predicted amino acid sequences of amoebic aSMases show a low homology with the eukaryotic aSMases reported, however, it presents the essential amino acid residues for the catalysis described for this type of enzymes [35]. There are no reports on aSMases sequences in other protozoa.

The amoebic aSMases have a signal peptide in the N-terminal region required for secretion, similar to other aSMases associated to lysosomes such as the human aSMase where a mutant lacking the signal peptide has no enzymatic activity and is not secreted [42,67]. The presence of cysteines in the C-terminal region suggests posttranslational processing related to the enzyme activation as reported in other aSMases [36,37]. Likewise, the amoebic aSMases have predicted residues for the coordination with cations, which orientates both the enzyme and the substrate in a suitable manner for the reaction as reported for *H*. *sapiens* [68,69], *C*. *elegans* [39] and *M*. *tuberculosis* [70] aSMases.

In this work, we focused on the *EhaSM6* gene, which exhibited the highest expression in *E*. *histolytica* trophozoites under basal conditions of growth. This gene codes for a functional protein as demonstrated in the protein expressed in *E*. *coli*, showing activity against sphingomyelin, being stimulated by Mg^2+^, inhibited by Co^2+^, and exhibited no effect by Zn^2+^. This is in contrast with other eukaryotic aSMases that require Zn^2+^ for activity [41,68]. The amoebic nSMases were stimulated by Mn^2+^ and partially inhibited by Zn^2+^ [71], while its counterpart in eukaryotes and prokaryotes responds to Mg^2+^ [72,73]. The aSMase activity was spontaneously secreted by *E*. *histolytica* trophozoites under standard culture conditions and exhibited the same effect of bivalent cation observed with the recombinant aSMase6. The aSMase activity released into the supernatant of trophozoites may be the contribution of the aSMases encoded by the six genes of *E*. *histolytica*. The transfected cell line of *E*. *histolytica* overexpressing the *EhaSM6*-HA gene exhibited a 2-fold increase of secreted aSMase activity suggesting that it is mainly due to the overexpression of the *EhaSM6* gene.

Reports of aSMases from mammalian cells indicate that they are lysosomal enzymes involved in the hydrolysis of sphingomyelin to produce ceramide, an important second messenger lipid associated with several cellular responses to stress, cell growth, differentiation, and apoptosis in eukaryotes [54]. In mammalian cells, the S-SMase is secreted spontaneously and its deficiency has been implicated in pathologies such as atherosclerosis [74,75], while the lysosomal aSMase has been detected after induction of stress and involvement in the repair of damage to the PM [44,76]. Therefore, it is of our interest to investigate if the *E*. *histolytica* trophozoites have a mechanism of plasma membrane repair mediated by aSMases which would allow it to survive the attack of lytic components of the host defense systems.

*E*. *histolytica* trophozoites of HM1-HA strain treated with the SLO showed an increase in the secreted aSMase activity, which in turn is 10-fold higher in the aSMase6 over-expressing strain, being these amoebae more resistant to the damage caused by the toxin than the control strain, showing a direct relationship between the level of aSMase secreted and amoebae viability. The aSMase6 over-expressing trophozoites exposed to another pore-forming molecules such as Magainin, β-Defensin 2 and human complement exhibited an significant increase in the secreted aSMase activity which correlated with higher amoebic viability in a Ca^+2^ dependent process. SLO molecules bind to cholesterol-containing target membranes to assemble, form rings that penetrate into the apolar domain of the lipid bilayer, resulting in the formation of pores of up to 30 nm in diameter. Membrane damage by SLO is basically analogous to channel formers, namely, the C5b-9 complement complex and some human antimicrobial peptides such as β-Defensin 2 [77]. Previous reports have shown in mammalian cells that after the damage to the PM by SLO, there is an intracellular flow of Ca^2+^ through the lesion that triggers the repair mechanism [8,44,78]. In *E*. *histolytica* trophozoites, extracellular Ca^2+^ seems to be an indispensable requirement for the secretion of aSMase activity in response to pore forming molecules exposition and for maintenance of viability, since in the absence of Ca^2+^, the activity of the secreted enzyme is null, and the viability is brought down completely. These results suggest that the aSMase6 of *E*. *histolytica* is secreted extracellularly during the induction of damage to the plasma membrane by SLO in a process Ca^2+^ dependent, and it is involved in the maintenance of amoebic viability. This process is related to the PM integrity restoration as shown for mammalian cells [44,47,79], but thus far, it has not been reported for *E*. *histolytica* trophozoites.

In mammalian cells, it has been described that after the increase of intracellular Ca^2+^, multiple calcium sensors such as synaptotagmin (Syt) VII, dysferlin and SNARE that promote lysosomal exocytosis towards the site of the lesion become involved in the PM repair [80–82]. The genome of *E*.*histolytica* encodes a large number of calcium-binding proteins, many of these proteins are unique to the amoebae, indicating that it has extensive Ca^+2^ signaling pathways [83], but only a few events mediated by this cation have been described. Even less known is the process of repair of damage to the membrane of *E*. *histolytica*. The SNARE complex and calcium-binding proteins, such as EhCaBP1 [84], could be involved in the exocytosis of lysosomes and the formation of endosomes in response to the increase in intracellular calcium concentration. There is evidence that some components of the SNARE complex can function in the trafficking of vesicles to the PM [85], as well as the EhRab7 and EhRab11 proteins that are involved in the biogenesis, acidification and trafficking of lysosomes, as well as in the trafficking of late endosomes and phagosomes [86–88]. It is likely that *E*. *histolytica* Rab proteins facilitate and regulate the kinetics of anchoring and pairing of the SNARE complex and promote exocytosis of lysosomes to the site of PM damage, similar to the already reported mechanism in mammalian cells [89,90], however, this still remains to be determined in *E*. *histolytica*.

Hydrogen peroxide, an important mediator of acute lipid oxidative injury, alters the fluidity and generates a leaky plasma membrane-associated with lipid peroxidation [91], also induced an increase of secreted aSMase in *E*. *histolytica*, but to a lesser extent. Hydrogen peroxide, a primary form of ROS in mammalian cells has been proposed as second mesangers in mammalian cells to mediate cellular responses, activating the aSMase translocation and activation [92]. The mechanism by which hydrogen peroxide induces aSMase secretion in *E*. *histolytica* remains to be investigated.

The C-terminal processing of human aSMases has been reported, which may exist associated with lysosomes or released extracellularly [93], which arise from the post-translational modifications during its vesicular trafficking and maturation [37,41,94]. To address the C-terminal processing and to explain the weak signal with anti-HA antibodies, the HA labeled aSMase6 was fused to the optimized Red Fluorescent Protein (RFP) for *E*. *histolytica* expression [60], and immunodetected with anti-HA antibodies. A protein corresponding to HA-RFP was detected corroborating the C-terminal processing. The C-terminal processing found in mammalian cells during the maturation of the enzyme not only modifies its molecular weight but also implying that this processing involves the elimination of the Cys 629 of the enzyme which increases its activity [36,59]. It is still necessary to deepen into the processing and activation of the aSMases of *E*. *histolytica*.

Confocal microscopy analysis of trophozoites exposed to SLO at early times showed that there is a migration of lysosomes to the PM and the formation of endosome structures in a "patch-like" arrangement which are transient structures that prevent the exit of the cytoplasmic components. Although these structures have been observed in mammalian cells after the induction of PM damage, they have not been well characterized. It has been suggested that they arise from the rapid and massive formation of endocytic vesicles, which accumulate near the site of the lesion [80,95–97]. The secretion of the aSMase by exocytosis of lysosomes is accompanied by the release of its content to the extracellular medium, determined by an increase in the activity of secreted CPs and generating an acidification of the extracellular medium on the periphery of the trophozoites (S7 Fig) after the SLO treatment allowing the aSMase activity on the sphingolipid substrate on the PM. The lysosomes are secretory vesicles that can release their content which may include lysosomal proteases [46] and transient acidification can be generated extracellularly at sites of lysosomal exocytosis [98,99]. Also, the role of released proteases from the lysosomes and their involvement in the resealing process of the plasma membrane needs to be elucidated, as described for mammalian cells [46]. The lysosome biogenesis that controls transport, maturation, and secretion of CPs and probably aSMases that could have an important role in the pathogenesis as well as housekeeping functions unrelated to parasitism and virulence in *E*. *histolytica* [100].

The increase in endosome formation after treatment with SLO is higher in aSMase6-HA overexpressing trophozoites compared with the control strain and trophozoites without treatment. This process has been observed after a few minutes of SLO exposure in NRK, HeLa or Jurkat cell-lines [44,101]. After massive endosomes formation, they fuse to lysosomes because they share fluorescence signal between lysosomes and endosomes following the vesicular traffic route, which has been described to be fully functional in amoebas, even though it lacks morphologically defined organelles such as the endoplasmic reticulum, Golgi apparatus, instead the amoebae have a high content of vesicles, many of which are associated with the functions of these organelles [102,103]. Exocytosis of aSMase by wounded cells promotes endocytosis and plasma membrane repair by the generation of the secondary messenger ceramide [44].

Sphingomyelin enriched lipid domains or ‘‘rafts” may serve as substrate pools for SMase-induced formation of ceramide microdomains that act as platforms from which these signal transduction cascades originate [104]. By analogy, aSMases could be involved in the production of ceramide associated with the stress responses in *E*. *histolytica* during the invasive process of the host [105,106], suggesting that conversion of plasma membrane sphingomyelin to ceramide by this lysosomal enzyme promotes lesion internalization.

TEM analysis shows the polarization of vesicles after exposure to SLO toxin, suggesting the participation of these vesicles in maintaining the integrity of the membrane at early damage-associated events. As the damage progresses, it is possible to observe an increase in polarized endosomes which internalize fragments of the damaged membrane, and the amoebae that repaired successfully the damage remain viable. In summary, the results presented here, suggest three main events after the amoebic membrane injury: first, the internalization of calcium through the lesion is important in the repair process that activates the lysosome exocytosis and aSMase release to initiate the repair process mechanism; second, the generation of patches formed from the fusion of lysosomes and endosomes at the damaged site, which momentarily prevent cell lysis, and third, the endocytosis-dependent generation of ceramide by the aSMase activity. The data presented here are consistent with the repair mechanism mediated by aSMase in mammalian cells [44,107]. This is the first report showing that *E*. *histolytica* has the machinery to repair PM damage mediated by aSMase6.

Secretion of aSMase has been detected in mammals after exposing the cells to different types of stress, in particular, it has been shown to be activated in response to damage to the PM, such as the caused by bacterial toxins, which generate small (0.5–5 nm) or large (20–100 nm) pores dependent on the concentration [44,47,108]; or viral, bacterial and parasitic pathogens (EBOV, *Neisseria gonorrhoeae*, *Staphylococcus aureus*, *Pseudomonas aeruginosa*, *Trypanosoma cruzi)*, which are associated with regions rich in cholesterol destabilizing the PM, thus generating lesions [109–113], leading to expect that amoebae could respond to PM damage caused by other molecules besides SLO toxin. During an intestinal human infection, several pore forming molecules are produced, where β-defensins are the most common [48]. The β-defensin 2 and the antimicrobial peptide magainin, isolated from *Xenopus laevis*, which is a cathelicidin, similar to the antimicrobial peptide LL-37, have been reported to have a lytic effect in *E*. *histolytica* trophozoites, interacting directly with the anionic phospholipids of the plasma membrane through the amphiphilic α-helix and forming permeable ion channels, resulting in depolarization, irreversible cytolysis and finally amoebic cell death [19,114,115]. Our results indicate that the overexpressing strain HM1-SM6HA is more resistant to damage with this peptide in comparison with the parental strain, which also correlated with higher secreted aSMase activity.

Interestingly, after SLO or β-Defensin 2 exposure there is an increase of gene expression of *EhaSM1*, *EhaSM2*, *EhaSM5* y *EhaSM6*, suggesting the potential participation of other members of aSMase gene family besides aSMase6 in the PM damage repair mechanism proposed in this work. Also, there is no change in gene expression for *EhaSM3* and *EhaSM4* genes after SLO treatment. The four *EhaSM* genes that respond to membrane damage, each one possesses a single calcineurin domain, while the two genes that do not respond to damage lack these domains. Calcineurin is a protein phosphatase regulated by Ca^2+^/calmodulin conserved in eukaryotes, and has been associated with stress response in yeast; this is activated when there is an increase in the concentration of cytosolic Ca^2+^ in response to internal or external signals, causing the activation of the Ca^2+^-calmodulin-binding domain and then subsequently binds to calcineurin, thus dephosphorylating the target proteins that modulate various biological processes that allow cell survival [116–118]. *E*. *histolytica* has genes that encode for calcineurin, but to date, there are no reports describing the processes in which they participate. Unlike what happens with pore-forming molecules, exposure to hydrogen peroxide increased the expression of the six genes that encode for aSMases in *E*. *histolytica*. In all the conditions evaluated, the *EhaSM6* gene exhibited the highest levels of induction. The above suggests that the expression of aSMasas in *E*. *histolytica* could be selective to different types of cellular stress.

The defensive response of the host is characterized by humoral and cellular immune reactions. The presence of amoebic trophozoites causes the infiltration of neutrophils, lymphocytes, and macrophage, and serum factors are amoebicidal through the activation of the alternative complement pathway [119]. While host cells elaborate diverse mechanisms for pathogen expulsion, amoebae have also developed complex strategies to modulate host immune response and facilitate their own survival [52,120]. In addition to virulence factors, there are other amoebic molecules, termed virulence determinants, that participate in the pathogenicity process by promoting the survival of parasites while confronting host defenses, allowing the parasites to harm the host [33]. It is not surprising that *E*. *histolytica* possess a mechanism of damage repair to the plasma membrane mediate by aSMase for maintaining trophozoites viability and to confront with various lytic agents such as antimicrobial peptides, bacterial toxins, and the complement system. Recently we reported that the nSMase3 of *E*. *histolytica* participate in hemolytic and cytotoxic activities, while, the nSMase1 and nSMase2 are involved in the cytopathic activity [121]. There are still new factors that remain to be elucidated and characterized to fully comprehend the virulence mechanism in this parasite. Further characterization of aSMases in *E*. *histolytica* is necessary to uncover their role in virulence as well as in cell signaling. The study of the relationship between aSMases and virulence is currently in process.

**Fig 11:**
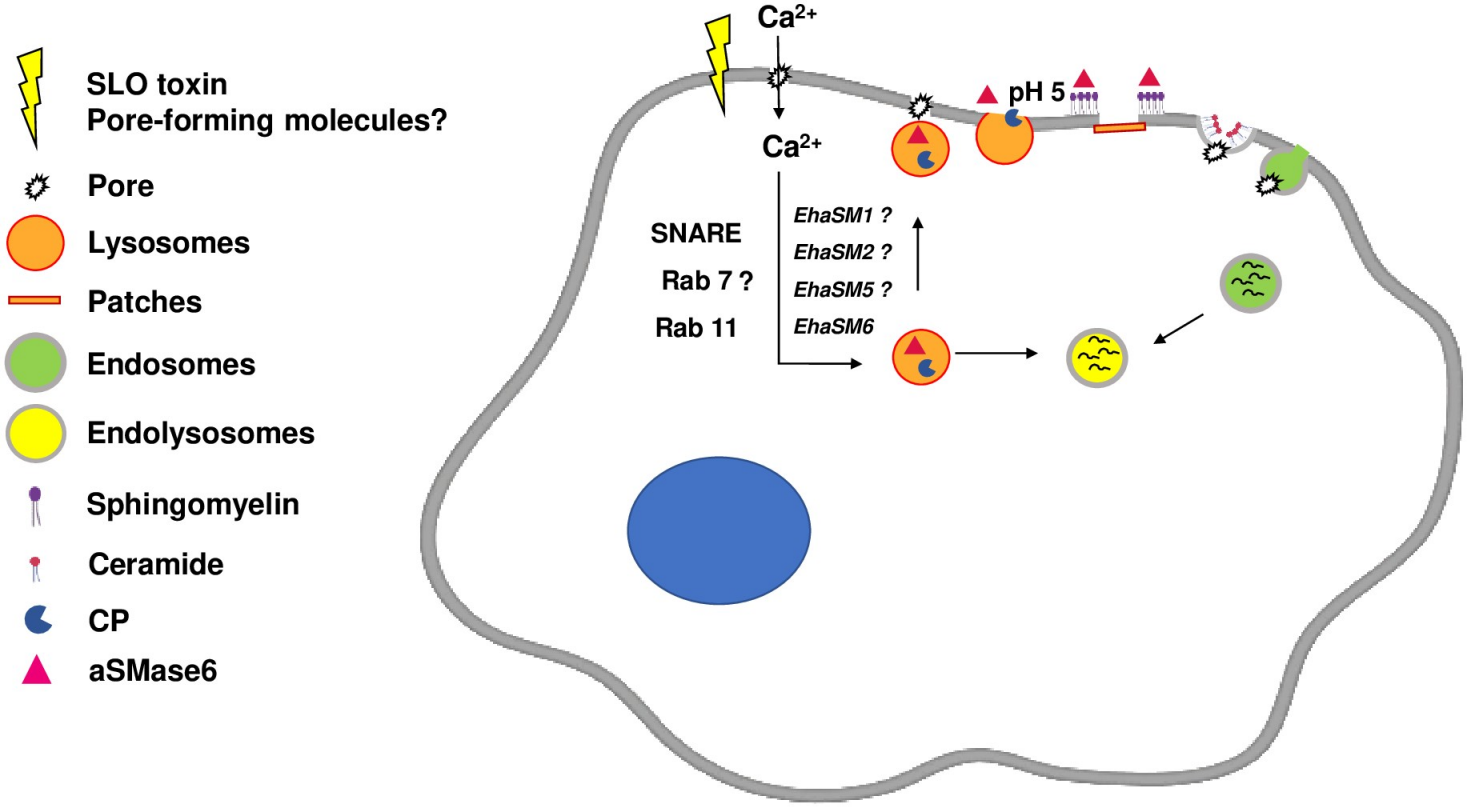
Model for plasma membrane repair mediated by secreted lysosomal aSMase6 in *E*. *histolytica*. The pore caused by SLO to the PM of *E*. *histolytica* allows the entry of extracellular Ca^2+^ into the cytoplasm. The elevation in the intracellular concentration of Ca^2+^ triggers the exocytosis of the lysosomes which discharge their content into the extracellular space, including the aSMase6. The aSMase6 hydrolyzes sphingomyelin membrane in ceramide, the latter favors the formation of endosomes that internalize the lesions.

Taking together all the results presented in this work, the damage of the trophozoites of *E*. *histolytica* with a sub-lethal concentration of SLO, induce the entry of Ca^2+^, which favors the migration of the lysosomes to the periphery of the cell, fuses with the plasma membrane and pour their content of aSMases to the outside of the cell. The secreted aSMases produce ceramide favoring the internalization of the lesion for its degradation in phagolysosomes. The pores generated by the PM damage are rapidly blocked by patch-like structures of lysosomes and endosomes that prevent the lysis of the trophozoite and immediately begin the internalizing the lesion. The aSMase6 overexpression favors the repair of the lesion and the survival of the trophozoites of *E*. *histolytica*. The plasma membrane damage repair mediated by aSMase in *E*. *histolytica* is summarized in Fig 11.

## Materials and methods

### Amoebae cell culture

Trophozoites of *E*. *histolytica* HM-1:IMSS and transfected trophozoites of the same strain were cultured under axenic conditions in Diamond’s TYI-S-33 medium supplemented with 10% adult bovine serum (Microlab Laboratories, Mexico) at 36 °C [122]. The transfectant strains were grown in the presence of G418 (Sigma-Aldrich, St. Louis, MO, USA), as a selective agent.

### Production of recombinant aSMase6 and activity determination

The encoding sequence of the amoebic aSMase6 (EHI_125660) was obtained by PCR from *E*. *histolytica* HM-1:IMSS cDNA using sense and antisense oligonucleotides containing the appropriate restriction sites (S1 Table). PCR product was cloned into pGEM-T (Promega) and subcloned into pRSET (Invitrogen). The resulting plasmids pRSET-aSM6 encoding the *EhaSM6* fused to a 6X histidine tag was verified by sequencing and used to transform *E*. *coli* BL21 AI cells (Invitrogen). For overexpressing the recombinant protein (rEhaSM6), BL21 AI culture was grown to a cell density of ∼0.4–0.6 OD 600 at 37 °C. To maximize the yield of recombinant soluble protein, *E*. *coli* cells were cultured for 4 h at 25 °C with 0.2% L-arabinose as an inductor.

Soluble rEhaSM6 was purified under native conditions for the detection of aSMase activity. Briefly, harvested *E*. *coli* BL21 AI cells were resuspended with 50 mM NaH_2_PO_4_, 300 mM NaCl, 10 mM imidazole (pH 8) at 2 ml per gram of wet weight. Lysozyme was added to the cell suspension at a final concentration of 10 mg/ml, and the mixture was incubated 30 min, followed by three freeze-thaw cycles. The cell lysate was centrifuged at 10,000×g for 20 min, and the rEhaSM6 was purified from the supernatant fraction using Ni-NTA Agarose (Qiagen) as directed by the manufacturer. The purified proteins were applied to a Hi-Trap desalting column (Amersham Pharmacia Biotech) and eluted with 100 mM Tris–HCl, 20 mM NaCl pH 7.5 buffer. The activity of the purified recombinant aSMase6 was determined using the Amplex Red Sphingomyelinase Assay Kit (Molecular Probes) as directed by the manufacturer. Briefly, a two-step assay was performed in 50 mM sodium acetate pH 5.0 containing sphingomyelin (0.5 mM), 2% Triton X-100, with 0.5 μg of the recombinant protein at 37 °C for 1 h. Subsequently, 100 mM Amplex Red reagent, 2 U mL^−1^ horseradish peroxidase, 0.2 U mL^−1^ choline oxidase and 8 U mL^−1^ alkaline phosphatase were added and mixed with 25 μL of 100 mM Tris–HCl (pH 8.0). After incubation for 20 min at 37°C; the fluorescence at 582 nm was measured, with excitation at 556 nm, using a Fluoroskan Ascent FL (Thermo Scientific) luminescence spectrometer. In all the assays, background from the enzyme-free controls were routinely subtracted from the activities of samples containing enzyme extracts.

### Generation of *E*. *histolytica* transfectant trophozoites overexpressing aSMase6

The *EhaSM6* coding sequence was PCR amplified from cDNA using sense and antisense oligonucleotides containing the appropriate restriction sites (S1 Table). A sequence tag consisting of three tandem repeats of hemagglutinin (HA) peptide was added at the C-terminus of aSMase by cloning the PCR products into pEhEx [123]. PCR fragments were digested with *Sma*I and *Xho*I and ligated into corresponding sites of the expression vector pEhEx. HM-1:IMSS trophozoites were transfected with the construct paSM6HA by liposome-mediated transfection as previously described [124,125]. *EhaSM6* over-expressing transfectants were selected with 40 μg/mL of G418 and maintained as stable cell lines. Over-expression of *EhaSM*6 gene was determined by quantitative real-time PCR (qPCR). Also, the paSMHA-RFP construction was designed to obtain the over-expressing strain of HA-tagged sphingomyelinase fused to a Red Fluorescent Protein (RFP) [60] in the C-terminal region (HM1-SM6HA-RFP).

### Assay of secreted aSMase activity in *E*. *histolytica*

Trophozoites in the exponential phase of growth were harvested. 3x10^5^ amoebae were placed per well of a 24-well cell culture plate and incubated at 37 °C in TYI-S-33 for 2 h. Adhered trophozoites were washed two times with Ca^2+^ free DMEM medium (Gibco, Life Technologies, Carlsbad, CA) and further incubated in 500 μL of DMEM medium containing 1.8 mM Ca^+2^ (Gibco, Life Technologies, Carlsbad, CA) at 37°C for the indicated time. After each incubation time, the collected supernatant was centrifugated at 15,890 x g for 3 min to remove any detached cells and the secreted aSMase activity was determined using the Amplex Red Sphingomyelinase Assay Kit, as described above.

### Assay for NADP^+^-dependent alcohol dehydrogenase in supernatants of *E*. *histolytica*

Supernatants from the secreted activity of aSMase were collected and the activity of alcohol dehydrogenase was evaluated as described previously [126]. Briefly, the buffer contains a 50 mM glycine/ NaOH buffer, pH 9.5, NADP^+^ 0–2 mM, 20 mM 2-propanol, and 150 μL of the sample is added to give a final volume of 200 μL. The reduction rate of NADP^+^ was evaluated at an absorbance of 340 nm at 25 °C for 60 min.

### Plasma membrane damage assay

Adhered amoebae in a 24 well cell culture plate were washed with DMEM medium without Ca^2+^, and then replenish with complete DMEM medium (1.8 mM Ca^2+^) pre-warmed at 37 °C and PM damage was performed by adding streptolysin-O (SLO) (Sigma-Aldrich), the antimicrobial peptides Defensin and Magainin II (Sigma-Aldrich), and Triton X-100 (Sigma-Aldrich). After exposing the trophozoites to various incubation times, the viability of trophozoites was determined (see below), and cell-free supernatants were collected and assayed for the secreted aSMase activity as described above.

### Susceptibility to complement-dependent lysis

Assays for susceptibility to human complement lysis were carried out with trophozoites during the logarithmic phase of growth. A previously published protocol was followed with some modifications [127,128]. Briefly, a total of 1×10^6^ trophozoites were incubated in buffer (PBS, 0.5 mM MgCl_2_, 1.25 mM CaCl_2_) with 50% normal human serum for 20 min at 37 °C. As a control for amoebic viability, trophozoites were incubated with heat-inactivated normal human serum (30 min at 56 °C). Trophozoites were centrifuged at 804 xg for 5 min, resuspended in 100 μl of PBS and stained with 0.2% Trypan blue dye (Microlab) to assess cell viability. The viability of the amoebae was measured by the exclusion of trypan blue dye. The average number of dead trophozoites that resulted from the incubation with heat-inactivated serum was subtracted from the average number of dead parasites incubated with normal human serum.

### Induction of oxidative stress in trophozoites of *E*. *histolytica* by the addition of H_2_O_2_

Adhered amoebae in a 24-well cell culture plate were washed with DMEM medium without Ca^2+^, then replenish with complete DMEM medium (1.8 mM Ca^2+^) pre-warmed at 37 °C and, different concentrations of H_2_O_2_ (0, 0.2, 0.5 and 1 mM) were added. The amoebas were incubated for 10 min to induce oxidative stress in the cells. After this time, the aSMase activity present in the supernatants and the viability of the trophozoites by trypan exclusion were evaluated.

### Cell viability assays

To evaluate the viability of trophozoites after SLO o Magainin II exposition, three methods were used: *i*) The viability of amoebae was measured by the exclusion of trypan blue dye. After plasma membrane damage, trophozoites were centrifuged at 804 xg for 5 min, resuspended in 100 μL of PBS and stained with 0.2% Trypan blue dye (Microlab). Viability was determined by counting the number of cells that did not incorporate the dye by light microscopy and counting 100 total trophozoites; *ii*) Live/Dead kit (Molecular Probes) was used according to the manufacturer's instructions. In brief, calcein-AM, and ethidium homodimer were added to trophozoites previously washed in PBS. Cells were incubated in these reagents for 10 min at room temperature and fluorescence was examined by epifluorescence microscopy (Zeiss Axioskop 40).

### Lysosome, endosome and nucleus labeling assay

For the staining of lysosomal compartments, live amoebae were growth in TYI-S-33 medium containing 2 μM Lysotracker Red DND-99 (Molecular Probes) for 14 h at 36°C. To label early endosomes, the amoebae stained with Lysotracker in exponential growth were harvested and transferred to coverslips placed in a 24 well cell culture plate and incubated at 37 °C for 2 h. The trophozoites were washed twice with Ca^2+^ free DMEM and further incubated in 500 μL of DMEM medium supplemented or not with SLO, and simultaneously exposed to 0.5 μg μL^-1^ fluorescein isothiocyanate (FITC)-labeled dextran (10,000 MW, Molecular Probes) followed by incubation at 37 °C for 1, 3 or 10 min. The cells were fixed for 10 min at room temperature with 3.7% paraformaldehyde in PBS and then incubated for 10 min at room temperature with 2 μM Höescht 33342 (Sigma-Aldrich, St. Louis, MO). Trophozoites were washed extensively with PBS, mounted using Vectashield (Vector Laboratories, Inc, USA) and recorded using a Zeiss LSM 700 confocal microscope.

### Determination of the released cysteine proteinase activity

Proteinase activity was measured using the synthetic peptide ZArg-Arg-pNA (Bachem) as a substrate [129]. 20 μl of cell-free supernatants collected from the secreted aSMase activity assays were combined with 180 μl of PBS and 2 μl of the 10 mM stock substrate for 2 h at 37 °C, reading every 15 min. The release of p-nitroaniline was measured in a microplate reader (Multiskan Go Thermo Scientific) at 405 nm. One unit of activity is defined as the number of micromoles of substrate hydrolyzed per min.

### Expression of another amoebic aSMases of the gene family in trophozoites that overexpress aSMase6

RNA was isolated from 5×10^6^ log-phase *E*. *histolytica* trophozoites using the Trizol reagent (Invitrogen) following the manufacturer’s protocol including DNase I (Qiagen) treatment. RNA was quantified, purity checked by absorbance at 260 and the 260/280 nm ratio respectively using a GeneQuant spectrophotometer (GE Healthcare), and integrity of isolated RNA was verified by gel electrophoresis. For first-strand cDNA synthesis, 3 μg of total RNA (DNA-free) isolated from amoebic trophozoites was reverse transcribed using Oligo (dT) and reverse transcriptase from the SuperScript II RT-system (Invitrogen) according to manufacturer’s instructions. For qPCR experiments, sense and antisense primers were designed (S1 Table) to amplify approximately 150 base pairs of the target gene sequences. qPCR was performed using the Step One Real-Time PCR System (Applied Biosystems) and Fast SYBR Green Master Mix (Applied Biosystems) following the manufacturer’s protocol. Relative quantification was carried out using the delta-delta Ct method [130] and *E*. *histolytica gapdh* gene transcript was used as house-keeping control. Two biological replicates were analyzed in triplicates.

### Immunoblotting assays

The trophozoites were harvested at the exponential phase of growth (5×10^6^ cells/ml) and washed twice with PBS pH 7.0. Trophozoites were re-suspended in lysis buffer (100 mM Tris–HCl pH 7.4 supplemented with 0.05 mM E64 and 1% Triton X-100) and disrupted with a hand homogenizer. Amoebic cell extracts were separated by SDS-PAGE and transferred to a nitrocellulose membrane (Hybond, Amersham Biosciences). Western blotting was performed using mouse anti-HA monoclonal antibody (1:500) (Invitrogen) as primary antibody and horseradish peroxidase-conjugated goat anti-mouse IgG (Amersham Pharmacia Biotech) as a secondary antibody and visualized using the alkaline phosphatase conjugate substrate kit (Bio-Rad). For equal amounts of protein, the concentration was determined by the DC Protein Assay (Bio-Rad).

### Transmission electron microscopy of amoebae treated with SLO

Trophozoites that had been exposed to SLO were washed once with PBS and twice with 0.1 M sodium cacodylate buffer at 37 °C and fixed for 3 h with 2.5% glutaraldehyde in 0.2 M sodium cacodylate buffer, pH 7.4. Fixed trophozoites were washed twice with 0.1 M sodium cacodylate buffer, post-fixed with 1.0% osmium tetroxide in 0.1M sodium cacodylate at 4 °C, dehydrated with ethanol at increasing concentrations and treated with propylene oxide. Trophozoites were embedded in EmBed 812 epoxy resins, polymerized blocks were cut using an ultramicrotome, and thin sections were stained with 2% uranyl acetate and 2% lead citrate. Trophozoites morphology was analyzed by transmission electron microscopy (TEM) with a JEM-1010 JEOL at 80 keV.

## Supporting information

S1 Fig Alignment between the predicted amino acid sequences of aSMases in *E*. *histolytica* and other organisms. Two aSMases of eukaryotes and two acid sphingomyelinase type phosphodiesterases were aligned with the sequences *EhaSM1*, *EhaSM2*, *EhaSM3*, *EhaSM4*, *EhaSM5* and *EhaSM6* of *E*. *histolytica*. The abbreviations of species are as follows: aSMhuman, aSMase from *Homo sapiens*, aSMmouse, aSMase from *Mus musculus*, aSM-Lhuman, phosphodiesterase type sphingomyelinase from *Homo sapiens* isoform eB; aSM-Lmouse, acid sphingomyelinase-type phosphodiesterase of *Mus musculus* isoform 3a 1. The alignment was done using CLUSTAL W (Thompson *et al*., 1994). Residues conserved in all sequences are indicated by asterisks. The important residues for catalysis are highlighted in gray. The predicted residues for metal coordination (▲), the conserved hydrophilic/aromatic cluster (●), and the conserved asa-type motif for substrate recognition "NX3CX3N" (◊) are indicated in the alignment. The cysteines involved in the disulfide bonds associated with the activity and secretion of the protein are indicated in the C-terminal sequences (○). The three disulfide bonds are indicated respectively as S1, S2, and S3. Calcineurin domain is indicated with a red line.
(TIF)

S2 Fig ADH and released aSMase activity by *E*. *histolytica* after SLO exposition. Activity of ADH and aSMase detected in supernatants collected after the amoebic exposition to different concentrations of SLO. The ADH activity detected in a total homogenate was used as 100% of activity. Different letters over the bars represent statistically significant differences at P ≤ 0.05 (Tukey–Kramer test).
(TIF)

S3 Fig Effect of Magainin and Triton X-100 on the secreted aSMase activity and viability of *E*. *histolytica* trophozoites. **A**. Trophozoites treated with Magainin. a. Secreted aSMase activity after 10 min of exposition to Magainin in complete DMEM medium with Ca^2+^ in HM1-HA and HM1-SM6HA strains. b. Viability of trophozoites after interaction with Magainin. **B**. Trophozoites treated with Triton X-100. c. Secreted aSMase activity after 5 min of exposition to Triton X-100 in complete DMEM medium with Ca^2+^ in HM1-HA and HM1-SM6HA strains. d. Viability of trophozoites after interaction with Triton X-100. The percentage (%) indicates the viability of trophozoites by the exclusion of trypan blue. Different letters over the bars represent statistically significant differences at P ≤ 0.05 (Tukey–Kramer test).
(TIF)

S4 Fig Transmission electron microscopy of *E*. *histolytica* trophozoites suspended and exposed to SLO. Trophozoites of HM1-HA (**A**) and HM1-SM6HA (**B**) were suspended and. exposed to 1.6 ng/μL of SLO for one min. Vesicles (v) and glycogen (g).
(TIF)

S5 Fig Secreted cysteine protease activity in transfectant trophozoites exposed to SLO. The specific activity of CPs was determined using the supernatants of HM1-HA and HM1-SM6HA strains, using the CP specific synthetic peptide z-Arg-Arg-pNA. The release of para-nitroaniline was quantified at 405 nm, with the specific activity expressed in μmol of hydrolyzed substrate per min per mL of amoebic supernatant. Trophozoites were exposed to 1.6 ng/μl of SLO for three min at 37 °C. Different letters over the bars represent statistically significant differences at P ≤ 0.05 (Tukey–Kramer test).
(TIF)

S6 Fig Secreted aSMase activity of trophozoites of HM1-SM6-HA-RFP strain. The aSMase activity was detected in cell-free supernatants of HM1-HA, HM1-SM6HA and HM1-SM6HA-RFP strains, collected after at 3 minutes in D-MEM medium at 37 ° C. Different letters over the bars represent statiscally significant differences at P ≤ 0.05 (Tukey–Kramer test).
(TIF)

S7 Fig Acidification of the extracellular medium on the periphery of the trophozoites. The secreted activity of aSMase was carried out for 10 min in DMEM medium (pH 7.0). The pH was determined in 100 μl fractions from top to the bottom. **A**. Strain HM1-HA. **B**. Strain HM1-HA exposed with SLO. **C**. Strain HM1-SM6HA. **D**. Strain HM1-SM6HA exposed to SLO. The trophozoites were treated with 1.6 ng/μL of SLO for three min at 37 °C.
(TIF)

S1 Table Primers used in this study for construct generation and quantitative and semi-quantitative PCR assays. (PDF)

S2 Table Expression levels of the EhaSM genes in the HM1-HA strain. (PDF)

S3 Table aSMase activity of recombinant EhaSM6 purified from *E*. *coli* and the effect of divalent cations. (PDF)

S4 Table Quantitative expression levels of EhaSM genes of *E*. *histolytica* in response to β-Defensin 2 exposition. (PDF)

S5 Table Quantification of endosomes present in trophozoites of strain HM1-HA and HM1-SM6HA of *E*. *histolytica* after treatment with SLO. (PDF)

## References

[1] Babiychuk EB, Monastyrskaya K, Potez S, Draeger A. Intracellular Ca2+ operates a switch between repair and lysis of streptolysin O-perforated cells. Cell Death Differ [Internet]. 2009;16(8):1126–34. Available from: 10.1038/cdd.2009.30 19325569

[2] Petrou T, Olsen HL, Thrasivoulou C, Masters JR, Ashmore JF, Ahmed A. Intracellular Calcium Mobilization in Response to Ion Channel Regulators via a Calcium-Induced Calcium Release Mechanism. J Pharmacol Exp Ther. 2016;360(2):378–87. 10.1124/jpet.116.236695 27980039 PMC5267512

[3] McNeil PL, Steinhardt RA. Plasma Membrane Disruption: Repair, Prevention, Adaptation. Annu Rev Cell Dev Biol [Internet]. 2003;19(1):697–731. Available from: http://www.annualreviews.org/doi/10.1146/annurev.cellbio.19.111301.140101 14570587 10.1146/annurev.cellbio.19.111301.140101

[4] Draeger A, Monastyrskaya K, Babiychuk EB. Plasma membrane repair and cellular damage control: The annexin survival kit. In: Biochemical Pharmacology [Internet]. Elsevier Inc.; 2011 p. 703–12. Available from: 10.1016/j.bcp.2010.12.027 21219882

[5] Futter CE, White IJ. Annexins and endocytosis. Traffic. 2007;8(8):951–8. 10.1111/j.1600-0854.2007.00590.x 17547702

[6] Potez S, Luginbühl M, Monastyrskaya K, Hostettler A, Draeger A, Babiychuk EB. Tailored protection against plasmalemmal injury by annexins with different Ca2+ sensitivities. In: Journal of Biological Chemistry. 2011 p. 17982–91. 10.1074/jbc.M110.187625 21454475 PMC3093872

[7] Draeger A, Schoenauer R, Atanassoff AP, Wolfmeier H, Babiychuk EB. Dealing with damage: Plasma membrane repair mechanisms. In: Biochimie [Internet]. Elsevier Masson SAS; 2014 p. 66–72. Available from: 10.1016/j.biochi.2014.08.008 25183513

[8] Idone V, Tam C, Goss JW, Toomre D, Pypaert M, Andrews NW. Repair of injured plasma membrane by rapid Ca2+ dependent endocytosis. In: Journal of Cell Biology. 2008 p. 905–14. 10.1083/jcb.200708010 18316410 PMC2265401

[9] Tam C, Idone V, Devlin C, Fernandes MC, Flannery A, He X, et al Exocytosis of acid sphingomyelinase by wounded cells promotes endocytosis and plasma membrane repair. J Cell Biol. 2010;189(6):1027–38. 10.1083/jcb.201003053 20530211 PMC2886342

[10] Holopainen JM, Medina OP, Metso AJ, Kinnunen PKJ. Sphingomyelinase Activity Associated with Human Plasma Low Density Lipoprotein POSSIBLE FUNCTIONAL IMPLICATIONS. J Biol Chem. 2000;275(22):16484–9. 10.1074/jbc.275.22.16484 10828058

[11] Gulbins E, Kolesnick R. Raft ceramide in molecular medicine. Oncogene. 2003;22(45 REV. ISS. 5):7070–7. 10.1038/sj.onc.1207146 14557812

[12] Organization WH. Strategies for the prevention of blindness in national programmes: a primary health care approach. World Health Organization; 1997.

[13] Stanley SL Jr. Amoebiasis. Lancet [Internet]. 2003 3 22;361(9362):1025–34. Available from: 10.1016/S0140-6736(03)12830-9 12660071

[14] Turkeltaub JA, McCarty TR III, Hotez PJ. The intestinal protozoa: emerging impact on global health and development. Curr Opin Gastroenterol. 2015;31(1):38–44. 10.1097/MOG.0000000000000135 25394233

[15] Guerrant RL, Brush J, Ravdin JI, Sullivan JA, Mandell GL. Interaction between Entamoeba histolytica and human polymorphonuclear neutrophils. J Infect Dis. 1981;143(1):83–93. 10.1093/infdis/143.1.83 6260869

[16] Denis M, Chadee K. Cytokine activation of murine macrophages for in vitro killing of Entamoeba histolytica trophozoites. Infect Immun. 1989;57(6):1750–6. 2542164 10.1128/iai.57.6.1750-1756.1989 PMC313351

[17] Lin J-Y, Seguin R, Keller K, Chadee K. Tumor necrosis factor alpha augments nitric oxide-dependent macrophage cytotoxicity against Entamoeba histolytica by enhanced expression of the nitric oxide synthase gene. Infect Immun. 1994;62(5):1534–41. 7513301 10.1128/iai.62.5.1534-1541.1994 PMC186349

[18] Cobo ER, He C, Hirata K, Hwang G, Tran U, Eckmann L, et al Entamoeba histolytica Induces Intestinal Cathelicidins but Is Resistant to Cathelicidin-Mediated Killing. 2012;143–9. 10.1128/IAI.05029-11 PMC3255679 22083705

[19] Ayala-Sumuano JT, Téllez-López VM, Domínguez-Robles M del C, Shibayama-Salas M, Meza I. Toll-like Receptor Signaling Activation by Entamoeba histolytica Induces Beta Defensin 2 in Human Colonic Epithelial Cells: Its Possible Role as an Element of the Innate Immune Response. In: PLoS Neglected Tropical Diseases. 2013. 10.1371/journal.pntd.0002083 PMC3585038 23469306

[20] Bohlson SS, Fraser DA, Tenner AJ. Complement proteins C1q and MBL are pattern recognition molecules that signal immediate and long-term protective immune functions. 2007;44:33–43. 10.1016/j.molimm.2006.06.021 16908067

[21] Costa CA, Nunes ÁC, Ferreira AJ, Gomes MA, Caliari M V. Entamoeba histolytica and E. dispar trophozoites in the liver of hamsters: In vivo binding of antibodies and complement. In: Parasites and Vectors. 2010 p. 1–10. 20338063 10.1186/1756-3305-3-23 PMC2861030

[22] Ravdin JI, John JE, Johnston LI, Innes DJ, Guerrant RL. Adherence of Entamoeba histolytica trophozoites to rat and human colonic mucosa. Infect Immun. 1985;48(2):292–7. 2580787 10.1128/iai.48.2.292-297.1985 PMC261303

[23] Petri WJ. Amebiasis and the Entamoeba histolytica Gal/GalNAc lectin: from lab bench to bedside. J Investig Med Off Publ Am Fed Clin Res. 1996;44(2):24. 8689398

[24] Mann BJ. Structure and function of the Entamoeba histolytica Gal/GalNAc lectin. In: International Review of Cytology. 2002 p. 59–80. 12049210 10.1016/s0074-7696(02)16003-7

[25] Macpherson AJ, McCoy KD, Johansen FE, Brandtzaeg P. The immune geography of IgA induction and function. Mucosal Immunol. 2008;1(1):11 10.1038/mi.2007.6 19079156

[26] Garcia-Nieto RM, Rico-Mata R, Arias-Negrete S, Avila EE. Degradation of human secretory IgA1 and IgA2 by Entamoeba histolytica surface-associated proteolytic activity. Parasitol Int. 2008;57(4):417–23. 10.1016/j.parint.2008.04.013 18571975

[27] Meyer M, Fehling H, Matthiesen J, Lorenzen S, Schuldt K, Bernin H, et al Overexpression of Differentially Expressed Genes Identified in Non-pathogenic and Pathogenic Entamoeba histolytica Clones Allow Identification of New Pathogenicity Factors Involved in Amoebic Liver Abscess Formation. PLoS Pathog. 2016;12(8):1–27. 10.1371/journal.ppat.1005853 PMC5004846 27575775

[28] Que X, Reed SL. The role of extracellular cysteine proteinases in pathogenesis of Entamoeba histolytica invasion. Parasitol Today. 1997;13(5):190–4. 15275090 10.1016/s0169-4758(97)01043-0

[29] Que X, Reed SL. Cysteine proteinases and the pathogenesis of amebiasis. Clin Microbiol Rev. 2000;13(2):196–206. 10.1128/cmr.13.2.196-206.2000 10755997 PMC100150

[30] Leippe M, Andrä J, Nickel R, Tannich E, Muller-Eberhard HJ. Amoebapores, a family of membranolytic peptides from cytoplasmic granules of Entamoeba histolytica: isolation, primary structure, and pore bacterial cytoplasmic membranes. Mol Microbiol. 1994;14(5):895–904. 10.1111/j.1365-2958.1994.tb01325.x 7715451

[31] Leippe M. Amoebapores. Parasitol Today. 1997;13(5):178–83. 15275088 10.1016/s0169-4758(97)01038-7

[32] Zhang X, Zhang Z, Alexander D, Bracha R, Mirelman D, Stanley SL. Expression of Amoebapores Is Required for Full Expression of Entamoeba histolytica Virulence in Amebic Liver Abscess but Is Not Necessary for the Induction of Inflammation or Tissue Damage in Amebic Colitis. Infect Immun. 2004;72(2):678–83. 10.1128/IAI.72.2.678-683.2004 14742508 PMC321641

[33] Anaya-Velázquez F, Padilla-Vaca F. Virulence of Entamoeba histolytica: a challenge for human health research. Future Microbiol. 2011;6(3):255–8. 10.2217/fmb.11.2 21449836

[34] Loftus B, Anderson I, Davies R, Alsmark UCM, Samuelson J, Amedeo P, et al The genome of the protist parasite Entamoeba histolytica. Nature. 2005;433(7028):865 10.1038/nature03291 15729342

[35] Seto M, Whitlow M, McCarrick MA, Srinivasan S, Zhu Y, Pagila R, et al A model of the acid sphingomyelinase phosphoesterase domain based on its remote structural homolog purple acid phosphatase. Protein Sci. 2004;13(12):3172–86. 10.1110/ps.04966204 15557261 PMC2287300

[36] Qiu H, Edmunds T, Baker-Malcolm J, Karey KP, Estes S, Schwarz C, et al Activation of human acid sphingomyelinase through modification or deletion of C-terminal cysteine. J Biol Chem. 2003;278(35):32744–52. 10.1074/jbc.M303022200 12801930

[37] Jenkins RW, Canals D, Idkowiak-Baldys J, Simbari F, Roddy P, Perry DM, et al Regulated Secretion of Acid Sphingomyelinase. In: Journal of Biological Chemistry. 2010 p. 35706–18. 10.1074/jbc.M110.125609 20807762 PMC2975195

[38] Wan Q, Schuchman EH. Biophysica. 1995;1270:207–10. 10.1016/0925-4439(95)00050-e 7727545

[39] L XH, H MO, K RN. Caenorhabditis elegans contains two distinct acid sphingomyelinases. J Biol Chem [Internet]. 1998;273(23):14374–9. Available from: http://www.wormbase.org/db/misc/paper?name=WBPaper00003086 9603947 10.1074/jbc.273.23.14374

[40] Kafadar KA, Cyert MS. Integration of stress responses: Modulation of calcineurin signaling in Saccharomyces cerevisiae by protein kinase A. In: Eukaryotic Cell. 2004 p. 1147–53. 10.1128/EC.3.5.1147-1153.2004 15470242 PMC522609

[41] Schissel SL, Keesler GA, Schuchman EH, Williams KJ, Tabas I. The cellular trafficking and zinc dependence of secretory and lysosomal sphingomyelinase, two products of the acid sphingomyelinase gene. J Biol Chem. 1998;273(29):18250–9. 10.1074/jbc.273.29.18250 9660788

[42] Jenkins RW, Canals D, Hannun YA. Roles and regulation of secretory and lysosomal acid sphingomyelinase. In: Cellular Signalling. 2009 p. 836–46. 19385042 10.1016/j.cellsig.2009.01.026 PMC3488588

[43] Johnson LR, Moss SB, Gerton GL. Maintenance of motility in mouse sperm permeabilized with streptolysin O. Biol Reprod. 1999;60(3):683–90. 10.1095/biolreprod60.3.683 10026116

[44] Tam C, Idone V, Devlin C, Fernandes MC, Flannery A, He X, et al Exocytosis of acid sphingomyelinase by wounded cells promotes endocytosis and plasma membrane repair. J Cell Biol. 2010;189(6):1027–38. 10.1083/jcb.201003053 20530211 PMC2886342

[45] Jaiswal JK, Andrews NW, Simon SM. Membrane proximal lysosomes are the major vesicles responsible for calcium-dependent exocytosis in nonsecretory cells. In: Journal of Cell Biology. 2002 p. 625–35. 10.1083/jcb.200208154 PMC2173094 12438417

[46] Castro-Gomes T, Corrotte M, Tam C, Andrews NW. Plasma membrane repair is regulated extracellularly by proteases released from lysosomes. In: PLoS ONE [Internet]. 2016 p. 1–26. Available from: 10.1371/journal.pone.0152583 PMC4814109 27028538

[47] Idone V, Tam C, Andrews NW. Two-way traffic on the road to plasma membrane repair. Trends Cell Biol. 2008;18(11):552–9. 10.1016/j.tcb.2008.09.001 18848451 PMC2593466

[48] Nuding S, Zabel LT, Enders C, Porter E, Fellermann K, Wehkamp J, et al Antibacterial activity of human defensins on anaerobic intestinal bacterial species: a major role of HBD-3. Microbes Infect. 2009;11(3):384–93. 10.1016/j.micinf.2009.01.001 19397883

[49] Selsted ME, Ouellette AJ. Mammalian defensins in the antimicrobial immune response. Nat Immunol. 2005;6(6):551 10.1038/ni1206 15908936

[50] Zasloff M. Antimicrobial peptides of multicellular organisms. Nature. 2002;415(6870):389 10.1038/415389a 11807545

[51] Tegla CA, Cudrici C, Patel S, Trippe R, Rus V, Niculescu F, et al Membrane attack by complement: the assembly and biology of terminal complement complexes. Immunol Res. 2011;51(1):45 10.1007/s12026-011-8239-5 21850539 PMC3732183

[52] Begum S, Quach J, Chadee K. Immune evasion mechanisms of Entamoeba histolytica: Progression to disease. In: Frontiers in Microbiology. 2015 p. 1–8. 26696997 10.3389/fmicb.2015.01394 PMC4678226

[53] Koley D, Bard AJ. Triton X-100 concentration effects on membrane permeability of a single HeLa cell by scanning electrochemical microscopy (SECM). Proc Natl Acad Sci. 2010;107(39):16783–7. 10.1073/pnas.1011614107 20837548 PMC2947864

[54] Clarke CJ, Hannun YA. Neutral sphingomyelinases and nSMase2: Bridging the gaps. Biochim Biophys Acta—Biomembr. 2006;1758(12):1893–901. 10.1016/j.bbamem.2006.06.025 16938269

[55] Nakada-Tsukui K, Tsuboi K, Furukawa A, Yamada Y, Nozaki T. A novel class of cysteine protease receptors that mediate lysosomal transport. Cell Microbiol. 2012;14(8):1299–317. 10.1111/j.1462-5822.2012.01800.x 22486861 PMC3465781

[56] Nakada-Tsukui K, Nozaki T. Molecular basis of the trafficking of cysteine proteases and other soluble lysosomal proteins in Entamoeba histolytica. In: Amebiasis. Springer; 2015 p. 279–304.

[57] Nakai K, Kanehisa M. A knowledge base for predicting protein localization sites in eukaryotic cells. Genomics. 1992;14(4):897–911. 1478671 10.1016/S0888-7543(05)80111-9 PMC7134799

[58] Nielsen H, Engelbrecht J, Brunak S, von Heijne G. Identification of prokaryotic and eukaryotic signal peptides and prediction of their cleavage sites. Protein Eng. 1997;10(1):1–6. 9051728 10.1093/protein/10.1.1

[59] Jenkins RW, Idkowiak-Baldys J, Simbari F, Canals D, Roddy P, Riner CD, et al A Novel Mechanism of Lysosomal Acid Sphingomyelinase Maturation. J Biol Chem. 2010;286(5):3777–88. 10.1074/jbc.M110.155234 21098024 PMC3030379

[60] Delgado-Galván CJ, Padilla-Vaca F, Montiel FBR, Rangel-Serrano Á, Paramo-Pérez I, Anaya-Velázquez F, et al Red fluorescent protein (DsRFP) optimization for Entamoeba histolytica expression. In: Experimental Parasitology. 2018 p. 86–92. 10.1016/j.exppara.2018.01.018 29476758

[61] Ravdin JI, Croft BY, Guerrant RL. Cytopathogenic mechanisms of Entamoeba histolytica. J Exp Med. 1980;152(2):377–90. 10.1084/jem.152.2.377 6249882 PMC2185944

[62] Tannicht E. Amoebic disease: Entamoeba histolytica and E. dispar: comparison of molecules considered important for host tissue destruction. Trans R Soc Trop Med Hyg. 1998;92(6):593–6. 10.1016/s0035-9203(98)90777-5 10326098

[63] Ralston KS, Petri WA Jr. Tissue destruction and invasion by Entamoeba histolytica. Trends Parasitol. 2011;27(6):254–63. 10.1016/j.pt.2011.02.006 21440507 PMC3104091

[64] Marie C, Petri WA Jr. Regulation of virulence of Entamoeba histolytica. Annu Rev Microbiol. 2014;68:493–520. 10.1146/annurev-micro-091313-103550 25002094 PMC9006484

[65] Moonah SN, Jiang NM, Petri WA. Host Immune Response to Intestinal Amebiasis. In: PLoS Pathogens. 2013 p. e1003489 10.1371/journal.ppat.1003489 23990778 PMC3749964

[66] Nakada-Tsukui K, Nozaki T. Immune response of amebiasis and immune evasion by Entamoeba histolytica. In: Frontiers in Immunology. 2016 p. 1–13. 27242782 10.3389/fimmu.2016.00175 PMC4863898

[67] Ferlinz K, Hurwitz R, Vielhaber G, Suzuki K, Sandhoff K. Occurrence of two molecular forms of human acid sphingomyelinase. Biochem J [Internet]. 1994;301 (Pt 3:855–62. Available from: http://www.ncbi.nlm.nih.gov/pubmed/8053910%0Ahttp://www.pubmedcentral.nih.gov/articlerender.fcgi?artid=PMC1137065 8053910 10.1042/bj3010855 PMC1137065

[68] Spence MW, Byers DM, Palmer FBSC, Cook HW. A new Zn2+-stimulated sphingomyelinase in fetal bovine serum. J Biol Chem. 1989;264(10):5358–63. 2538416

[69] Tam C, Idone V, Devlin C, Fernandes MC, Flannery A, He X, et al Exocytosis of acid sphingomyelinase by wounded cells promotes endocytosis and plasma membrane repair. In: Journal of Cell Biology. 2010 p. 1027–38. 10.1083/jcb.201003053 20530211 PMC2886342

[70] Vargas-Villarreal J, Mata-Cárdenas BD, Deslauriers M, Quinn FD, Castro-Garza J, Martínez-Rodrĺguez HG, et al Identification of acidic, alkaline, and neutral sphingomyelinase activities in Mycobacterium tuberculosis. Med Sci Monit [Internet]. 2003;9(6):BR225–30. Available from: http://www.ncbi.nlm.nih.gov/pubmed/12824945 12824945

[71] Mendoza-Macías CL, Barrios-Ceballos MP, Anaya-Velázquez F, Nakada-Tsukui K, Nozaki T, Padilla-Vaca F. Entamoeba histolytica: Molecular cloning and characterization of a novel neutral sphingomyelinase. Exp Parasitol [Internet]. 2010;125(3):279–85. Available from: 10.1016/j.exppara.2010.02.001 20138872

[72] Rao BG, Spence MW. Sphingomyelinase activity at pH 7.4 in human brain and a comparison to activity at pH 5.0. J Lipid Res. 1976;17:506–15. 9463

[73] Matsuo Y, Yamada A, Tsukamoto K, Tamura HO, Ikezawa H, Nakamura H, et al A distant evolutionary relationship between bacterial sphingomyelinase and mammalian DNase I. Protein Sci. 1996;5(12):2459–67. 10.1002/pro.5560051208 8976554 PMC2143316

[74] Xu XX, Tabas I. Sphingomyelinase enhances low density lipoprotein uptake and ability to induce cholesteryl ester accumulation in macrophages. J Biol Chem. 1991;266(36):24849–58. 1761578

[75] Leventhal AR, Chen W, Tall AR, Tabas I. Acid Sphingomyelinase-deficient Macrophages Have Defective Cholesterol Trafficking and Efflux. J Biol Chem. 2001;276(48):44976–83. 10.1074/jbc.M106455200 11579092

[76] Kolesnick RN, Goñi FM, Alonso A. Compartmentalization of ceramide signaling: physical foundations and biological effects. J Cell Physiol. 2000;184(3):285–300. 10.1002/1097-4652(200009)184:3<285::AID-JCP2>3.0.CO;2-3 10911359

[77] Bhakdi S, Tranum-Jensen J, Sziegoleit A. Mechanism of membrane damage by streptolysin-O. Infect Immun. 1985;47(1):52–60. 3880730 10.1128/iai.47.1.52-60.1985 PMC261464

[78] Tam C, Flannery AR, Andrews N. Live Imaging Assay for Assessing the Roles of Ca^2+^ and Sphingomyelinase in the Repair of Pore-forming Toxin Wounds. In: Journal of Visualized Experiments. 2013 p. 1–8. 10.3791/50531 PMC3856312 23995606

[79] Walev I, Bhakdi SC, Hofmann F, Djonder N, Valeva A, Aktories K, et al Delivery of proteins into living cells by reversible membrane permeabilization with streptolysin-O. Proc Natl Acad Sci. 2001;98(6):3185–90. 10.1073/pnas.051429498 11248053 PMC30628

[80] McNeil PL, Vogel SS, Miyake K, Terasaki M. Patching plasma membrane disruptions with cytoplasmic membrane. In: Journal of cell science [Internet]. 2000 p. 1891–902. Available from: http://www.ncbi.nlm.nih.gov/pubmed/10806100 10806100 10.1242/jcs.113.11.1891

[81] Reddy A, Caler E V., Andrews NW. Plasma membrane repair is mediated by Ca2+-regulated exocytosis of lysosomes. Cell. 2001;106(2):157–69. 10.1016/s0092-8674(01)00421-4 11511344

[82] Rao SK, Huynh C, Proux-Gillardeaux V, Galli T, Andrews NW. Identification of SNAREs Involved in Synaptotagmin VII-regulated Lysosomal Exocytosis. J Biol Chem. 2004;279(19):20471–9. 10.1074/jbc.M400798200 14993220

[83] Bhattacharya A, Padhan N, Jain R, Bhattacharya S. Calcium-binding proteins of Entamoeba histolytica. Arch Med Res. 2006;37(2):221–5. 10.1016/j.arcmed.2005.10.002 16380322

[84] Sahoo N, Labruyère E, Bhattacharya S, Sen P, Guillén N, Bhattacharya A. Calcium binding protein 1 of the protozoan parasite Entamoeba histolytica interacts with actin and is involved in cytoskeleton dynamics. J Cell Sci. 2004;117(16):3625–34. 15252130 10.1242/jcs.01198

[85] Herman E, Siegesmund MA, Bottery MJ, Van Aerle R, Shather MM, Caler E, et al Membrane Trafficking Modulation during Entamoeba Encystation. In: Scientific Reports [Internet]. Springer US; 2017 p. 1–17. Available from: 10.1038/s41598-017-12875-6 28993644 PMC5634486

[86] McGugan GC, Temesvari LA. Characterization of a Rab11-like GTPase, EhRab11, of Entamoeba histolytica. Mol Biochem Parasitol. 2003;129(2):137–46. 12850258 10.1016/s0166-6851(03)00115-4

[87] Saito-nNkano Y, Mitra BN, Nakada-tsukui K, Sato D, Nozaki T. Two Rab7 isotypes, Eh Rab7A and Eh Rab7B, play distinct roles in biogenesis of lysosomes and phagosomes in the enteric protozoan parasite Entamoeba histolytica. In: Cellular Microbiology. 2007 p. 1796–808. 10.1111/j.1462-5822.2007.00915.x 17359234

[88] Smith SS, Guillen N. Organelles and Trafficking in Entamoeba histolytica In: Structures and Organelles in Pathogenic Protists. Springer; 2010 p. 149–73.

[89] Roche PA. Intracellular protein traffic in lymphocytes: “How do I get there from here?” Immunity. 1999;11(4):391–8. 10549621 10.1016/s1074-7613(00)80114-4

[90] Cheng X, Zhang X, Yu L, Xu H. Calcium signaling in membrane repair. In: Seminars in cell & developmental biology. Elsevier; 2015 p. 24–31. 10.1016/j.semcdb.2015.10.031 PMC4681278 26519113

[91] Block ER. Hydrogen peroxide alters the physical state and function of the plasma membrane of pulmonary artery endothelial cells. J Cell Physiol. 1991;146(3):362–9. 10.1002/jcp.1041460305 1902481

[92] Li X, Gulbins E, Zhang Y. Oxidative stress triggers Ca2+-dependent lysosome trafficking and activation of acid sphingomyelinase. Cell Physiol Biochem. 2012;30(4):815–26. 10.1159/000341460 22890197 PMC3777434

[93] Schissel SL, Schuchman EH, Williams KJ, Tabas I. Zn2+-stimulated sphingomyelinase is secreted by many cell types and is a product of the acid sphingomyelinase gene. J Biol Chem. 1996;271(31):18431–6. 10.1074/jbc.271.31.18431 8702487

[94] Lansmann S, Ferlinz K, Hurwitz R, Bartelsen O, Glombitza G, Sandhoff K. Purification of acid sphingomyelinase from human placenta: Characterization and N-terminal sequence. FEBS Lett. 1996;399(3):227–31. 10.1016/s0014-5793(96)01331-2 8985151

[95] Miyake K, McNeil PL. Vesicle accumulation and exocytosis at sites of plasma membrane disruption. J Cell Biol. 1995;131(6):1737–45. 8557741 10.1083/jcb.131.6.1737 PMC2120668

[96] McNeil PL, Kirchhausen T. An emergency response team for membrane repair. Nat Rev Mol cell Biol. 2005;6(6):499 10.1038/nrm1665 15928713

[97] Andrews NW, Corrotte M, Castro-Gomes T. Above the fray: Surface remodeling by secreted lysosomal enzymes leads to endocytosis-mediated plasma membrane repair. In: Seminars in Cell and Developmental Biology [Internet]. Elsevier Ltd; 2015 p. 10–7. Available from: 10.1016/j.semcdb.2015.09.022 PMC4679444 26433178

[98] Baron R, Neff L, Louvard D, Courtoy PJ. Cell-mediated extracellular acidification and bone resorption: evidence for a low pH in resorbing lacunae and localization of a 100-kD lysosomal membrane protein at the osteoclast ruffled border. J Cell Biol. 1985;101(6):2210–22. 10.1083/jcb.101.6.2210 3905822 PMC2114017

[99] Andrews NW. Regulated secretion of conventional lysosomes. Trends Cell Biol. 2000;10(8):316–21. 10884683 10.1016/s0962-8924(00)01794-3

[100] Nozaki T, Nakada-Tsukui K. Membrane trafficking as a virulence mechanism of the enteric protozoan parasite Entamoeba histolytica. Parasitol Res. 2006;98(3):179–83. 10.1007/s00436-005-0079-6 16374616

[101] Shakor ABA, Atia MM, Kwiatkowska K, Sobota A. Cell surface ceramide controls translocation of transferrin receptor to clathrin-coated pits. Cell Signal. 2012;24(3):677–84. 10.1016/j.cellsig.2011.10.016 22101012

[102] López RS. El proceso de N-glicosilación de proteínas en Entamoeba histolytica. Rev Latinoam Microbiol. 2006;48(2):70–2. 17578075

[103] Perdomo D, Aït-Ammar N, Syan S, Sachse M, Jhingan GD, Guillén N. Cellular and proteomics analysis of the endomembrane system from the unicellular Entamoeba histolytica. J Proteomics. 2015;112:125–40. 10.1016/j.jprot.2014.07.034 25109464

[104] Dobrowsky RT. Sphingolipid signalling domains. Floating on rafts or buried in caves? Cell Signal. 2000;12(2):81–90. 10679576 10.1016/s0898-6568(99)00072-8

[105] Olivos-García A, Saavedra E, Nequiz M, Santos F, Luis-García ER, Gudiño M, et al The oxygen reduction pathway and heat shock stress response are both required for Entamoeba histolytica pathogenicity. Curr Genet. 2016;62(2):295–300. 10.1007/s00294-015-0543-5 26589893

[106] Pineda E, Perdomo D. Entamoeba histolytica under Oxidative Stress: What Countermeasure Mechanisms Are in Place? Cells [Internet]. 2017;6(4):44 Available from: http://www.mdpi.com/2073-4409/6/4/44 10.3390/cells6040044 PMC5755502 29160807

[107] Schuchman EH. Acid sphingomyelinase, cell membranes and human disease: Lessons from Niemann-Pick disease. FEBS Lett. 2010;584(9):1895–900. 10.1016/j.febslet.2009.11.083 19944693

[108] Los FCO, Randis TM, Aroian R V., Ratner AJ. Role of Pore-Forming Toxins in Bacterial Infectious Diseases. Microbiol Mol Biol Rev [Internet]. 2013;77(2):173–207. Available from: http://mmbr.asm.org/cgi/doi/10.1128/MMBR.00052-12 23699254 10.1128/MMBR.00052-12 PMC3668673

[109] Grassmé H, Gulbins E, Brenner B, Ferlinz K, Sandhoff K, Harzer K, et al Acidic sphingomyelinase mediates entry of N. gonorrhoeae into nonphagocytic cells. Cell. 1997;91(5):605–15. 10.1016/s0092-8674(00)80448-1 9393854

[110] Grassme H, Jendrossek V, Riehle A, Von Kürthy G, Berger J, Schwarz H, et al Host defense against Pseudomonas aeruginosa requires ceramide-rich membrane rafts. Nat Med. 2003;9(3):322 10.1038/nm823 12563314

[111] Fernandes MC, Cortez M, Flannery AR, Tam C, Mortara RA, Andrews NW. Trypanosoma cruzi subverts the sphingomyelinase-mediated plasma membrane repair pathway for cell invasion. J Exp Med. 2011;208(5):909–21. 10.1084/jem.20102518 21536739 PMC3092353

[112] Miller ME, Adhikary S, Kolokoltsov AA, Davey RA. Ebolavirus Requires Acid Sphingomyelinase Activity and Plasma Membrane Sphingomyelin for Infection. In: Journal of Virology. 2012 p. 7473–83. 10.1128/JVI.00136-12 22573858 PMC3416309

[113] Li C, Wu Y, Riehle A, Orian-Rousseau V, Zhang Y, Gulbins E, et al Regulation of Staphylococcus aureus infection of macrophages by CD44, reactive oxygen species, and acid sphingomyelinase. Antioxid Redox Signal. 2018;28(10):916–34. 10.1089/ars.2017.6994 28747072

[114] Huang CM, Chen HC, Zierdt CH. Magainin analogs effective against pathogenic protozoa. Antimicrob Agents Chemother. 1990;34(9):1824–6. 10.1128/aac.34.9.1824 2285300 PMC171939

[115] Rivas L, Luque-Ortega JR, Andreu D. Amphibian antimicrobial peptides and Protozoa: Lessons from parasites. Biochim Biophys Acta—Biomembr [Internet]. 2009;1788(8):1570–81. Available from: 10.1016/j.bbamem.2008.11.002 19046939

[116] Rusnak F, Mertz P. Calcineurin: form and function. Physiol Rev. 2000;80(4):1483–521. 10.1152/physrev.2000.80.4.1483 11015619

[117] Sugiura R, Sio SO, Shuntoh H, Kuno T. Calcineurin phosphatase in signal transduction: Lessons from fission yeast. Genes to Cells. 2002;7(7):619–27. 12081640 10.1046/j.1365-2443.2002.00557.x

[118] Park HS, Chow EWL, Fu C, Soderblom EJ, Moseley MA, Heitman J, et al Calcineurin Targets Involved in Stress Survival and Fungal Virulence. PLoS Pathog. 2016;12(9):1–20. 10.1371/journal.ppat.1005873 PMC5017699 27611567

[119] Sacks D, Sher A. Evasion of innate immunity by parasitic protozoa. Nat Immunol. 2002;3(11):1041 10.1038/ni1102-1041 12407413

[120] Guo X, Houpt E, Petri WA Jr. Crosstalk at the initial encounter: interplay between host defense and ameba survival strategies. Curr Opin Immunol. 2007;19(4):376–84. 10.1016/j.coi.2007.07.005 17702556 PMC2679172

[121] Luz U-R, Fátima R-M, Sairy A-G, Itzel P-P, Ángeles R-S, Reyes-Cortes R, et al Contribution of neutral sphingomyelinases to in vitro virulence of Entamoeba histolytica. Exp Parasitol. 2018;194:38–44. 10.1016/j.exppara.2018.09.015 30253133

[122] Diamond LS, Harlow DR, Cunnick CC. A new medium for the axenic cultivation of Entamoeba histolytica and other Entamoeba. Trans R Soc Trop Med Hyg. 1978;72(4):431–2. 10.1016/0035-9203(78)90144-x 212851

[123] Saito-Nakano Y, Yasuda T, Nakada-Tsukui K, Leippe M, Nozaki T. Rab5-associated vacuoles play a unique role in phagocytosis of the enteric protozoan parasite Entamoeba histolytica. J Biol Chem. 2004;279(47):49497–507. 10.1074/jbc.M403987200 15347665

[124] Nozaki T, Asai T, Sanchez LB, Kobayashi S, Nakazawa M, Takeuchi T. Characterization of the Gene Encoding Serine Acetyltransferase, a Regulated Enzyme of Cysteine Biosynthesis from the Protist ParasitesEntamoeba histolytica and Entamoeba dispar REGULATION AND POSSIBLE FUNCTION OF THE CYSTEINE BIOSYNTHETIC PATHWAY IN ENTAM. J Biol Chem. 1999;274(45):32445–52. 10.1074/jbc.274.45.32445 10542289

[125] Mendoza-Macías CL, Barrios-Ceballos MP, Anaya-Velázquez F, Nakada-Tsukui K, Nozaki T, Padilla-Vaca F. Entamoeba histolytica: Molecular cloning and characterization of a novel neutral sphingomyelinase. Exp Parasitol [Internet]. 2010;125(3):279–85. Available from: 10.1016/j.exppara.2010.02.001 20138872

[126] Lo HS, Chang CJ. Purification and properties of NADP-linked, alcohol dehydrogenase from Entamoeba histolytica. J Parasitol. 1982;68(3):372–7. 6284905

[127] Calderón J, Tovar R. Loss of susceptibility to complement lysis in Entamoeba histolytica HM1 by treatment with human serum. Immunology. 1986;58(3):467 2874111 PMC1453474

[128] Rastew E, Morf L, Singh U. Entamoeba histolytica rhomboid protease 1 has a role in migration and motility as validated by two independent genetic approaches. Exp Parasitol. 2015;154:33–42. 10.1016/j.exppara.2015.04.004 25889553 PMC4444385

[129] Leippe M, Sievertsen HJ, Tannich E, Horstmann RD. Spontaneous release of cysteine proteinases but not of pore-forming peptides by viable Entamoeba histolytica. Parasitology. 1995;111(5):569–74. 8559589 10.1017/s0031182000077040

[130] Livak KJ, Schmittgen TD. Analysis of relative gene expression data using real-time quantitative PCR and the 2− ΔΔCT method. methods. 2001;25(4):402–8. 10.1006/meth.2001.1262 11846609

