# Supplementary figures and images for: Plasma membrane damage repair is mediated by an acid sphingomyelinase in Entamoeba histolytica
Source: PLoS Pathog. 2019 Aug 28;15(8):e1008016. doi: 10.1371/journal.ppat.1008016 (PMC6713333; doi:10.1371/journal.ppat.1008016)

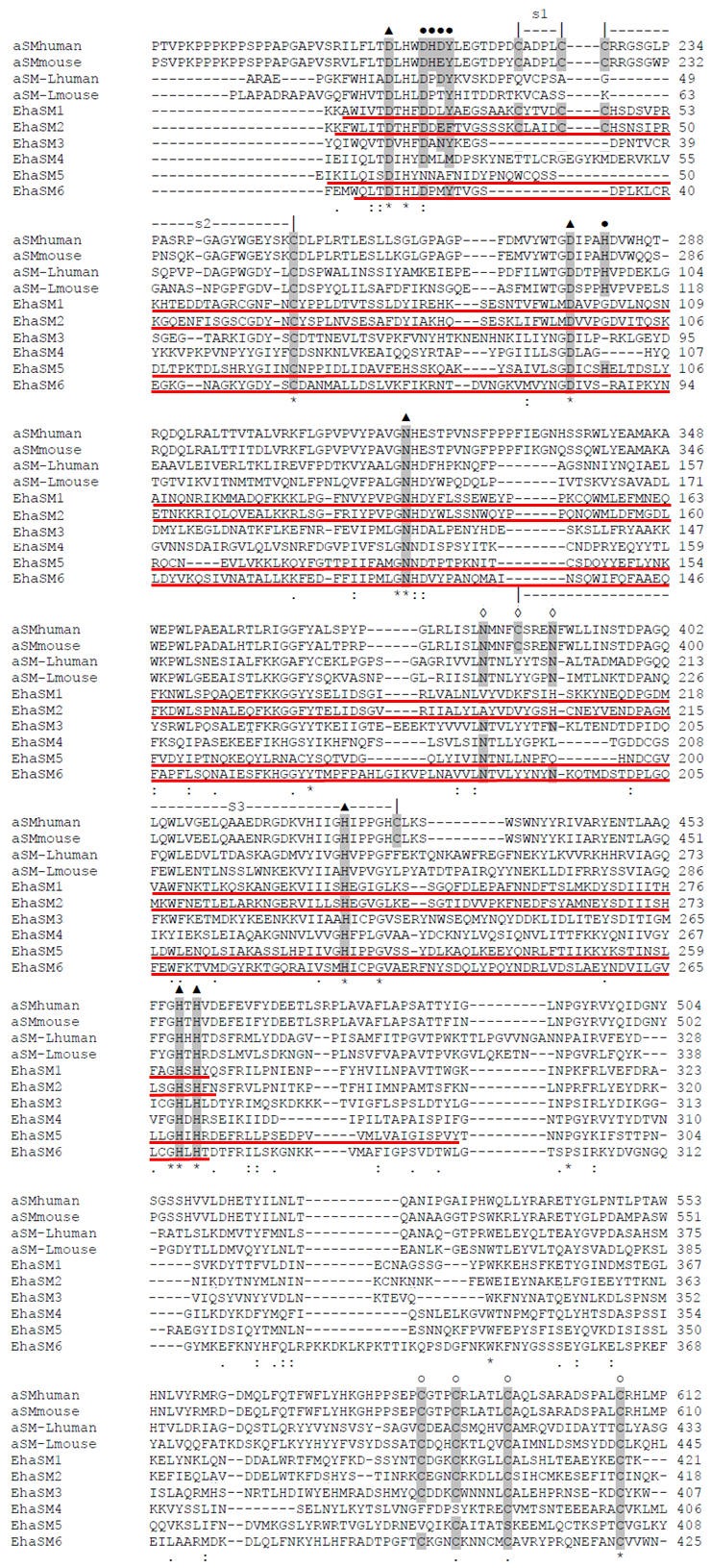

Supplement: S1 Fig — Two aSMases of eukaryotes and two acid sphingomyelinase type phosphodiesterases were aligned with the sequences EhaSM1, EhaSM2, EhaSM3, EhaSM4, EhaSM5 and EhaSM6 of E. histolytica. The abbreviations of species are as follows: aSMhuman, aSMase from Homo sapiens, aSMmouse, aSMase from Mus musculus, aSM-Lhuman, phosphodiesterase type sphingomyelinase from Homo sapiens isoform eB; aSM-Lmouse, acid sphingomyelinase-type phosphodiesterase of Mus musculus isoform 3a 1. The alignment was done using CLUSTAL W (Thompson et al., 1994). Residues conserved in all sequences are indicated by asterisks. The important residues for catalysis are highlighted in gray. The predicted residues for metal coordination (▲), the conserved hydrophilic/aromatic cluster (●), and the conserved asa-type motif for substrate recognition "NX3CX3N" (◊) are indicated in the alignment. The cysteines involved in the disulfide bonds associated with the activity and secretion of the protein are indicated in the C-terminal sequences (○). The three disulfide bonds are indicated respectively as S1, S2, and S3. Calcineurin domain is indicated with a red line. (TIF) [file ppat.1008016.s001.tif]

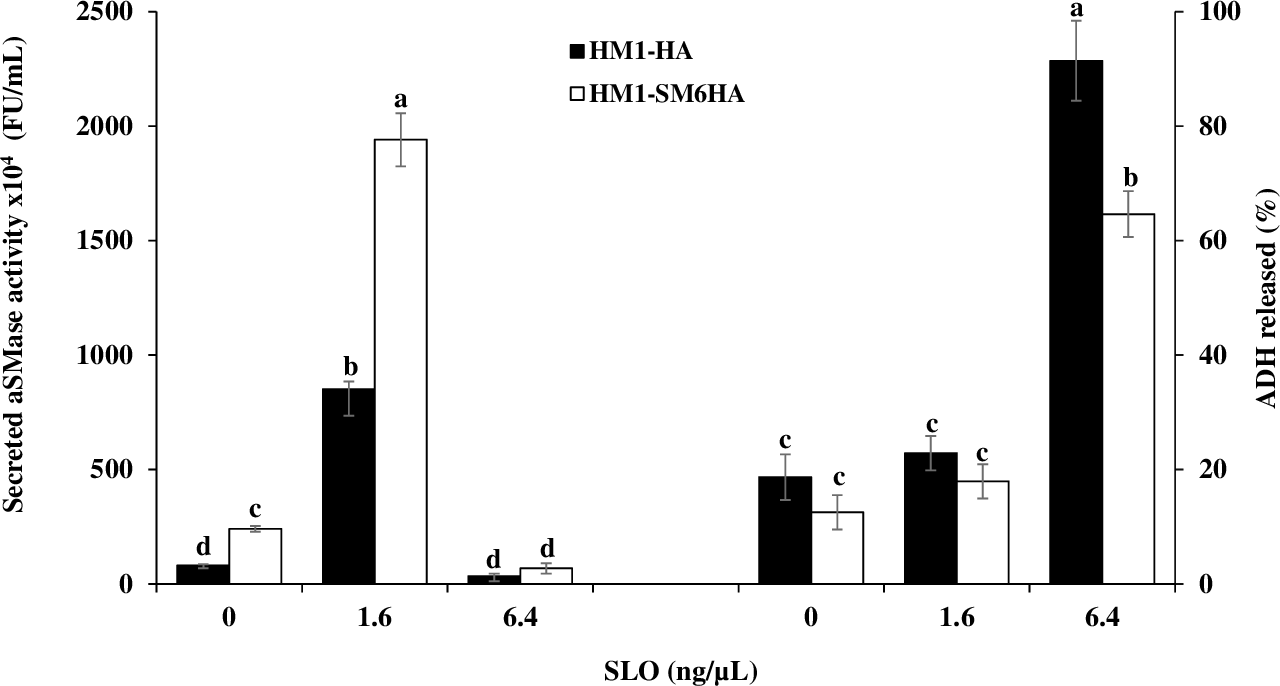

Supplement: S2 Fig — Activity of ADH and aSMase detected in supernatants collected after the amoebic exposition to different concentrations of SLO. The ADH activity detected in a total homogenate was used as 100% of activity. Different letters over the bars represent statistically significant differences at P ≤ 0.05 (Tukey–Kramer test). (TIF) [file ppat.1008016.s002.tif]

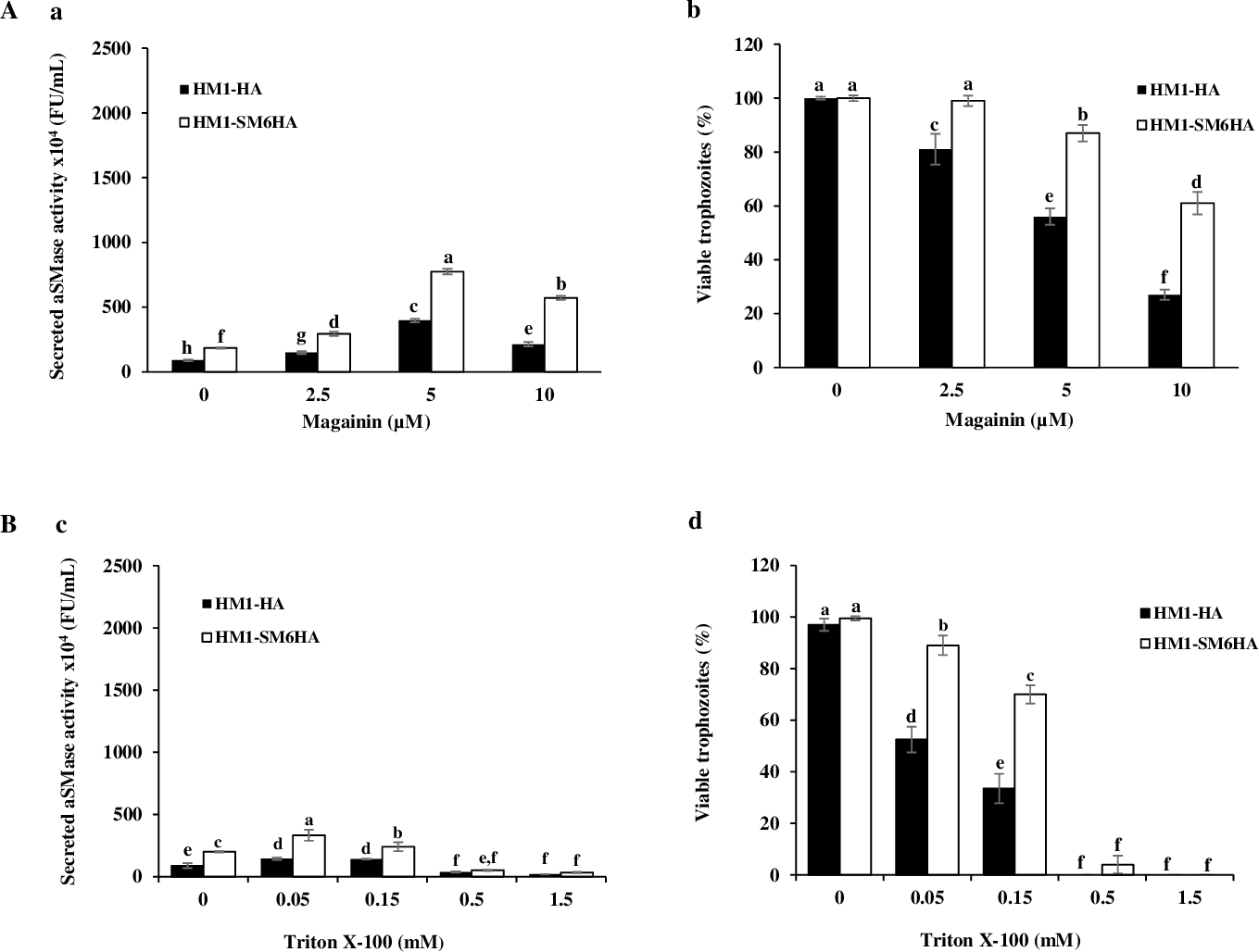

Supplement: S3 Fig — A. Trophozoites treated with Magainin. a. Secreted aSMase activity after 10 min of exposition to Magainin in complete DMEM medium with Ca2+ in HM1-HA and HM1-SM6HA strains. b. Viability of trophozoites after interaction with Magainin. B. Trophozoites treated with Triton X-100. c. Secreted aSMase activity after 5 min of exposition to Triton X-100 in complete DMEM medium with Ca2+ in HM1-HA and HM1-SM6HA strains. d. Viability of trophozoites after interaction with Triton X-100. The percentage (%) indicates the viability of trophozoites by the exclusion of trypan blue. Different letters over the bars represent statistically significant differences at P ≤ 0.05 (Tukey–Kramer test). (TIF) [file ppat.1008016.s003.tif]

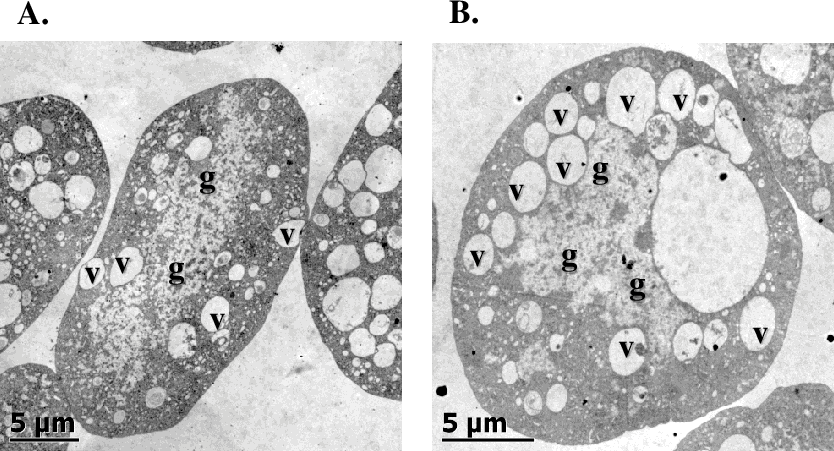

Supplement: S4 Fig — Trophozoites of HM1-HA (A) and HM1-SM6HA (B) were suspended and. exposed to 1.6 ng/μL of SLO for one min. Vesicles (v) and glycogen (g). (TIF) [file ppat.1008016.s004.tif]

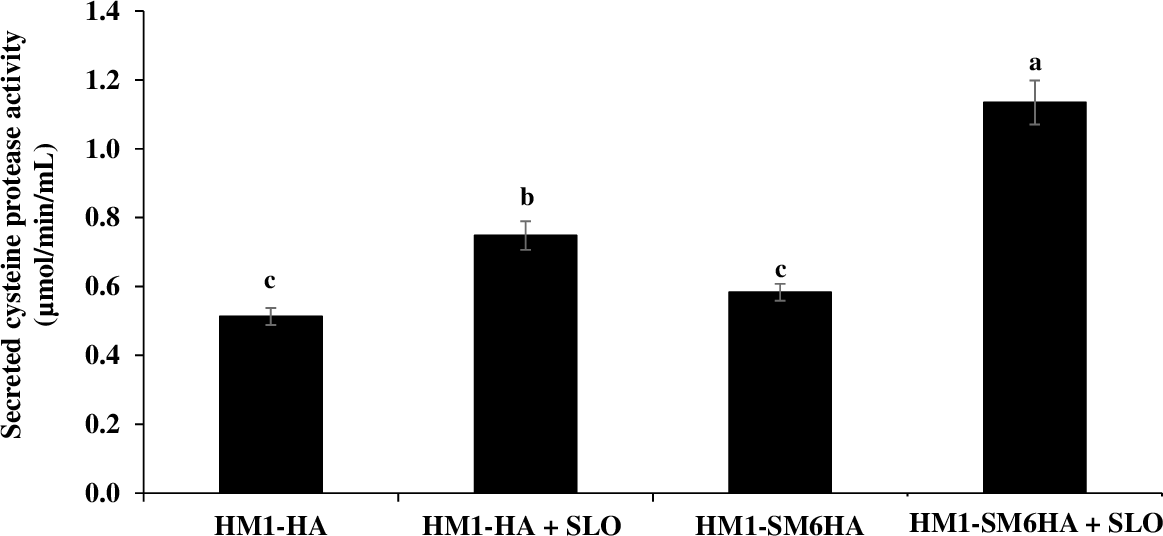

Supplement: S5 Fig — The specific activity of CPs was determined using the supernatants of HM1-HA and HM1-SM6HA strains, using the CP specific synthetic peptide z-Arg-Arg-pNA. The release of para-nitroaniline was quantified at 405 nm, with the specific activity expressed in μmol of hydrolyzed substrate per min per mL of amoebic supernatant. Trophozoites were exposed to 1.6 ng/μl of SLO for three min at 37 °C. Different letters over the bars represent statistically significant differences at P ≤ 0.05 (Tukey–Kramer test). (TIF) [file ppat.1008016.s005.tif]

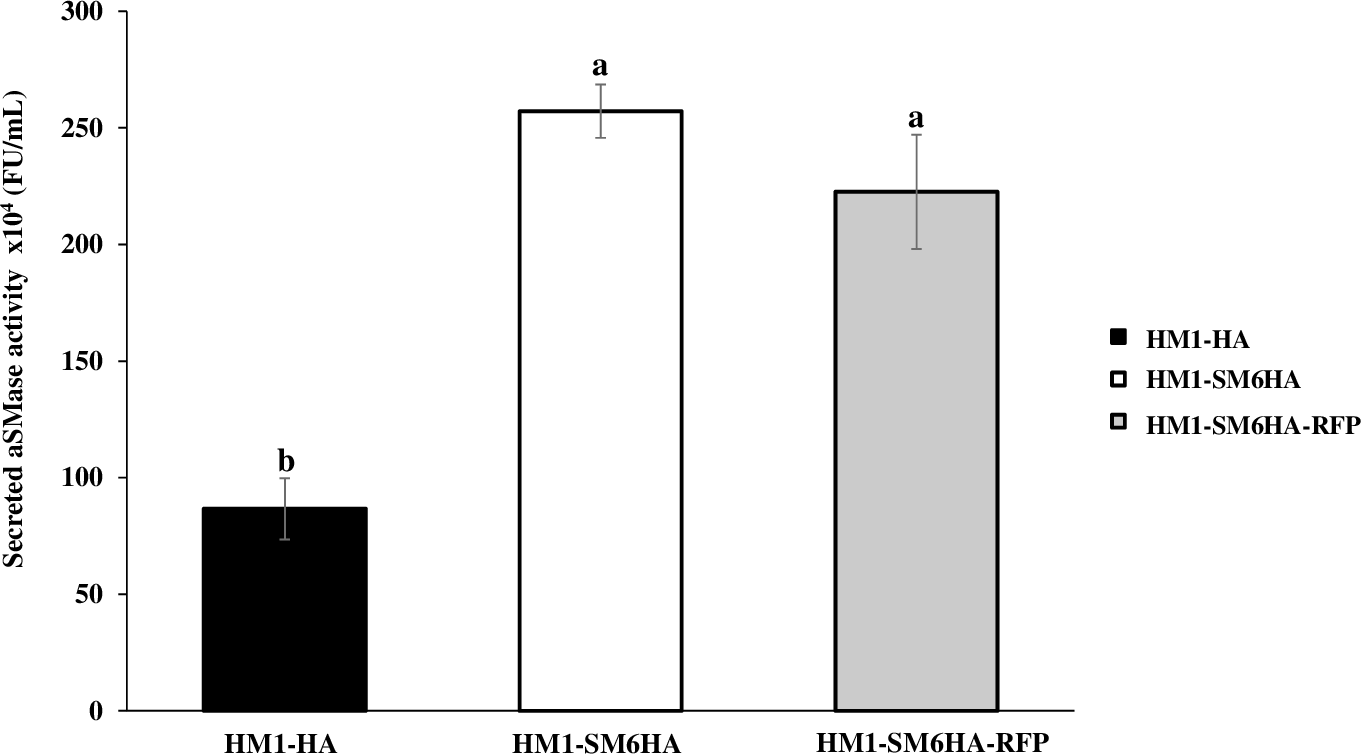

Supplement: S6 Fig — The aSMase activity was detected in cell-free supernatants of HM1-HA, HM1-SM6HA and HM1-SM6HA-RFP strains, collected after at 3 minutes in D-MEM medium at 37 ° C. Different letters over the bars represent statiscally significant differences at P ≤ 0.05 (Tukey–Kramer test). (TIF) [file ppat.1008016.s006.tif]

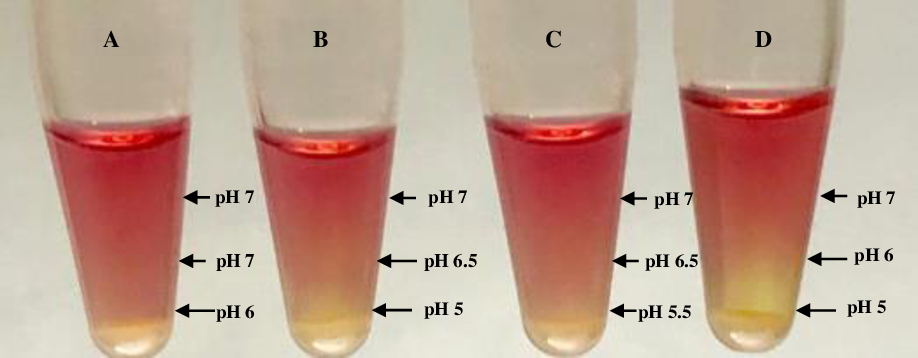

Supplement: S7 Fig — The secreted activity of aSMase was carried out for 10 min in DMEM medium (pH 7.0). The pH was determined in 100 μl fractions from top to the bottom. A. Strain HM1-HA. B. Strain HM1-HA exposed with SLO. C. Strain HM1-SM6HA. D. Strain HM1-SM6HA exposed to SLO. The trophozoites were treated with 1.6 ng/μL of SLO for three min at 37 °C. (TIF) [file ppat.1008016.s007.tif]
