# Supplementary material for: Plasma membrane damage repair is mediated by an acid sphingomyelinase in Entamoeba histolytica
Source: PLoS Pathog. 2019 Aug 28;15(8):e1008016. doi: 10.1371/journal.ppat.1008016 (PMC6713333; doi:10.1371/journal.ppat.1008016)
Supplement: S1 Table — (PDF) [file ppat.1008016.s008.pdf]

S1 Table. Primers used in this study for construct generation and quantitative and semi-quantitative PCR assays.

Access	Target gene name	Sequence	Orientation	PCR
XM_651327	Glyceraldehyde-3-phosphate dehydrogenase	5´- TACCGTCCACAGACAATTCG -3´	Forward	Q[a]
XM_651327	Glyceraldehyde-3-phosphate dehydrogenase	5´- TAGGCTGGATCTCTTTCAGC -3´	Reverse	Q
XM_646365	Acid Sphingomyelinase 1	5´- TCGATTGTTGCCATTCAGAC -3´	Forward	Q
XM_646365	Acid Sphingomyelinase 1	5´- CTCCAGGAACAGCATCCATT -3´	Reverse	Q
XM_643770	Acid Sphingomyelinase 2	5´- AGATGGCTTCCACAAAGTGC -3´	Forward	Q
XM_643770	Acid Sphingomyelinase 2	5´- TTGATCAATTGGGTCTGTATC -3´	Reverse	Q
XM_650953	Acid Sphingomyelinase 3	5´- GATTGGCTTTCTCCAAATGC -3´	Forward	Q
XM_650953	Acid Sphingomyelinase 3	5´- TCCTGCTGGATCATTTTC -3´	Reverse	Q
XM_646625	Acid Sphingomyelinase 4	5´- GGACATTTCCCACTTGGTGT -3´	Forward	Q
XM_646625	Acid Sphingomyelinase 4	5´- GAACGATGGTCATGTCCAAAT -3´	Reverse	Q
XM_644619	Acid Sphingomyelinase 5	5´- CCATTCCAGCACAATGACTG -3´	Forward	Q
XM_644619.1	Acid Sphingomyelinase 5	5´- CACGGTGAATATGCCCAAGT -3´	Reverse	Q
XM_651421	Acid Sphingomyelinase 6	5´- GGCATTTATTGGACCGTCTG -3´	Forward	Q
XM_651421	Acid Sphingomyelinase 6	5´- CTTGTCCATTTCCAACATCG -3´	Reverse	Q
XM_645264; XM_649267	Glyceraldehyde-3-phosphate dehydrogenase	5´- ATTAAAATATGATACCGTCCACAGA -3´	Forward	SQ[b]
XM_645264; XM_649267	Glyceraldehyde-3-phosphate dehydrogenase	5´- TGAGTAGCAGTAGTAGCATGAATAGTT -3´	Reverse	SQ
XM_651421	Acid Sphingomyelinase 6	5´- GTCTGTAGACACATGGTTAGGAAC -3´	Forward	SQ
	Acid Sphingomyelinase 6	5´- GTCTTGGATATCTCACAGCACAC -3´	Reverse	SQ
	Acid Sphingomyelinase 6 with site *Bgl*ll	5´- GAAGATCTATGGTATTTCAAATA -3´	Forward	Cloning
	Acid Sphingomye<linase 6 with site *Bgl*ll	5´- GAAGATCTATTCCAACTACACA-3´	Reverse	Cloning
	Acid Sphingomyelinase 6 Stop mutagenesis	5´- AATTGTGTAGTTTGGAATTATCCATATGAT GTTCCAGATTATGCTCTCGAGTTGAACTCTTC -3´	Forward	Site directed mutagenesis
	Acid Sphingomyelinase 6 Stop mutagenesis	5´- GAAGAGTTCAACTCGAGAGCATAATCTGGA ACATCATATGGATAATTCCAAACTACACAATT -3´	Reverse	Site directed mutagenesis

[a]Q = Quantitative real time PCR

[b]SQ = Semi-quantitative PCR
