# Supplementary material for: Plasma membrane damage repair is mediated by an acid sphingomyelinase in Entamoeba histolytica
Source: PLoS Pathog. 2019 Aug 28;15(8):e1008016. doi: 10.1371/journal.ppat.1008016 (PMC6713333; doi:10.1371/journal.ppat.1008016)
Supplement: S2 Table — (PDF) [file ppat.1008016.s009.pdf]

S2 Table. Expression levels of the *EhaSM* genes in the HM1-HA strain.

Strain / ***EhaSM* gene**	**HM1:IMSS**
EhaSM1	0.093 ± 0.017
EhaSM2	0.004 ± 0.001
EhaSM3	0.01 ± 0.004
EhaSM4	0.26 ± 0.046
EhaSM5	0.11 ± 0.009
EhaSM6	1

Data were normalized using the $\Delta\Delta^{CT}$ method against the gene *EhaSM6*.
