# Supplementary material for: Plasma membrane damage repair is mediated by an acid sphingomyelinase in Entamoeba histolytica
Source: PLoS Pathog. 2019 Aug 28;15(8):e1008016. doi: 10.1371/journal.ppat.1008016 (PMC6713333; doi:10.1371/journal.ppat.1008016)
Supplement: S3 Table — (PDF) [file ppat.1008016.s010.pdf]

S3 Table. aSMase activity of recombinant EhaSM6 purified from *E. coli* and the effect of divalent cations.

Recombinant EhaSM6 activity (%)*				
**mM**	**Mg^{+2}**	**Co^{+2}**	**Mn^{+2}**	**Zn^{+2}**
0	100	100	100	100
0.5	ND	28.06	ND	ND
1	ND	6.01	ND	ND
5	160.14	0	101.49	84.99
10	181.14	0	105.31	81.37
20	212. 16	0	104.68	91.75

* The activity is shown as relative values of the control activity determined in the absence of the cations. Control protein extract from a strain bearing the empty plasmid did not show aSMase activity.

ND (Not determined)
