# Supplementary material for: Plasma membrane damage repair is mediated by an acid sphingomyelinase in Entamoeba histolytica
Source: PLoS Pathog. 2019 Aug 28;15(8):e1008016. doi: 10.1371/journal.ppat.1008016 (PMC6713333; doi:10.1371/journal.ppat.1008016)
Supplement: S4 Table — (PDF) [file ppat.1008016.s011.pdf]

S4 Table. Quantitative expression levels of *EhaSM* genes of *E. histolytica* in response to β-Defensin 2 exposition.

Strain / *EhaSM genes*	HM1-HA	HM1-HA + Defensin*	HM1-SM6HA	HM1-SM6HA + Defensin*
EhaSM1	1.0	3.94 ± 0.25	1.23 ± 0.05	3.05 ± 0.11
EhaSM2	1.0	4.37 ± 0.15	1.16 ± 0.29	4.98 ± 0.14
EhaSM3	1.0	1.01 ± 0.11	1.06 ± 0.49	1.23 ± 0.17
EhaSM4	1.0	1.90 ± 0.03	1.45 ± 0.19	1.64 ± 0.28
EhaSM5	1.0	4.17 ± 0.12	1.15 ± 0.09	4.79 ± 0.33
EhaSM6	1.0	6.14 ± 0.39	6.91 ± 0.68	9.93 ± 0.12

Data were normalized using the ΔΔCT method against the housekeeping gene *Ehgapdh*. The control HM1-HA strain expression ratio is set to 1.0 and that the values represent the fold of over-expression.

* Trophozoites treated with 30 ng/mL of β-Defensin 2 for 30 min at 37 °C.
