# Supplementary material for: Plasma membrane damage repair is mediated by an acid sphingomyelinase in Entamoeba histolytica
Source: PLoS Pathog. 2019 Aug 28;15(8):e1008016. doi: 10.1371/journal.ppat.1008016 (PMC6713333; doi:10.1371/journal.ppat.1008016)
Supplement: S5 Table — (PDF) [file ppat.1008016.s012.pdf]

S5 Table. Quantification of endosomes present in trophozoites of strain HM1-HA and HM1-SM6HA of *E. histolytica* after treatment with SLO.

Strain	Average number of endosomes per amoeba (Confocal)*	Relative fluorescence units (494 - 521 nm) **
**HM1-HA**	2.8	108.7
**HM1-HA + SLO**	8.2	489.5
**HM1-SM6HA**	5.2	261.9
**HM1-SM6HA + SLO**	29.8	1557.1

The trophozoites were treated with 1.6 ng/µL of SLO, for three min., at 37 ºC.

* Endosomes were counted in 250 trophozoites.

** Amoebae lysates after SLO treatment and then fluorescence quantification.
